# Prognostic Significance of the Pretreatment Neutrophil‐to‐Lymphocyte Ratio and Tumor‐Associated Neutrophils in Head and Neck Squamous Cell Carcinoma: Systematic Review and Meta‐Analysis

**DOI:** 10.1111/jop.70110

**Published:** 2025-12-30

**Authors:** Ahana Kapuge Dona Varuni Yashodha Ratnayake, Aini Hyytiäinen, Georgia Vasiliki Gkountana, Meri Torri, Ahmed Al‐Samadi

**Affiliations:** ^1^ Department of Oral and Maxillofacial Diseases Clinicum, University of Helsinki Helsinki Finland; ^2^ Institute of Dentistry, School of Medicine, Faculty of Health Sciences, University of Eastern Finland Kuopio Finland

**Keywords:** head and neck squamous cell carcinoma, meta‐analysis, neutrophil‐lymphocyte ratio, prognosis, systematic review, tumor‐associated neutrophils

## Abstract

**Objectives:**

The prognostic significance of the neutrophil‐to‐lymphocyte ratio (NLR) and tumor‐associated neutrophils (TANs) in head and neck squamous cell carcinoma (HNSCC) remains a subject of considerable research interest. This systematic review and meta‐analysis aimed to evaluate the relationship between pretreatment NLR and TANs and survival outcomes in patients with HNSCC.

**Methods:**

The systematic literature search was conducted using PubMed, Scopus, Web of Science, and the Cochrane Library. Studies that documented pretreatment NLR in peripheral blood or TANs and their association with disease‐specific survival (DSS), progression‐free survival (PFS), overall survival (OS), disease‐free survival (DFS), and recurrence‐free survival (RFS) were included. Meta‐analyses were performed using the R “meta” package. Subgroup analyses were performed based on tumor site and NLR thresholds. Heterogeneity was assessed using Cochran's Q and Higgins' I^2^ statistics, while publication bias was evaluated using Egger's test and funnel plots.

**Results:**

A total of 100 studies comprising 26 324 patients were included. The hazard ratios (HR) for OS, PFS, DFS, DSS, and RFS were 1.88, 1.95, 1.85, 2.16, and 1.11, respectively, in univariate analysis. Multivariate analysis supported these findings and showed similar trends. Subgroup analyses indicated that high NLR consistently predicted poor OS across all studied tumor sites and NLR thresholds. Elevated densities of TANs, particularly CD15^+^ and CXCR4^+^ subsets, were linked to poorer cancer‐specific survival and OS, respectively.

**Conclusion:**

This meta‐analysis indicates that elevated pretreatment NLR, particularly above four, may serve as a significant prognostic marker of poor survival outcomes in patients with HNSCC.

## 1 Introduction

Head and neck cancer (HNC) is the world's sixth most prevalent cancer, with approximately 950 000 new cases in 2022 [1]. Ninety percent of HNC is head and neck squamous cell carcinoma (HNSCC), which arises in the squamous epithelium of the head and neck region, including the oral and nasal cavities, nasopharynx, oropharynx, hypopharynx, and larynx [2]. The tumor microenvironment (TME) of HNSCC, as well as other solid cancers, is comprised of various immune cells, including neutrophils, lymphocytes, NK cells, and macrophages, along with non‐immune stroma cells such as cancer‐associated fibroblasts (CAFs) and extracellular components. The type, density, and localization of immune cells within the TME have been linked to the clinical outcome of various malignancies [3, 4].

Neutrophils, which are the most abundant myeloid cells in human blood, demonstrate diverse plasticity when they infiltrate into tumors. Tumor‐associated neutrophils (TANs) can either suppress or promote cancer progression, exhibiting anti‐tumor (N1) or protumor (N2) phenotypes, in a similar way to macrophages [5, 6]. Concerning the anti‐tumor role, TANs are capable of releasing cytotoxic granules, including defensins and cathelicidins, which can destroy cancer cells [7]. Additionally, neutrophil‐derived reactive oxygen species can induce tumor cell apoptosis, senescence, or cell cycle arrest [8]. Conversely, in their protumor phenotype, TANs can contribute to cancer progression by promoting angiogenesis, facilitating immune evasion, enhancing tumor cell proliferation, and suppressing the anti‐tumor immune response through the release of immunosuppressive cytokines and pro‐angiogenic factors [9, 10, 11, 12]. It has been shown that high infiltration of TANs intratumorally in several cancers, including HNSCC, results in poor clinical outcomes such as increased tumor aggressiveness and decreased patient survival rates [13].

Currently, the key parameters for assessing the patients' prognosis and staging of HNSCC include tumor size and depth, and the presence of nodal and distant metastases according to the American Joint Committee on Cancer (AJCC). However, as these staging systems continue to be updated, regular revisions and improved parameters are required to ensure the best and most up‐to‐date patient management [14]. Although molecular markers have been investigated for prognostic use in cancer patients, their reliability is still limited due to tumor heterogeneity [15]. Moreover, their accessibility and availability are still restricted in many parts of the world. Therefore, the development of novel, easily measurable hematological biomarkers is crucial for enabling better patient stratification and facilitating personalized treatment strategies [16]. Increasing evidence suggests that multiple hematologic inflammatory markers can predict the prognosis of several types of cancer, such as C‐reactive protein (CRP), neutrophil‐to‐lymphocyte ratio (NLR), platelet‐to‐lymphocyte ratio, and lymphocyte‐to‐monocyte ratio [17, 18]. Among these, the NLR in peripheral blood is one of the most widely studied, since it can be easily calculated from routine blood test results. The prognostic significance of the NLR has been reported in multiple meta‐analyses spanning different cancers [19, 20, 21].

This systematic review and meta‐analysis aimed to evaluate the prognostic value of NLR and TANs in HNSCC patients in terms of overall survival (OS), disease‐free survival (DFS), progression‐free survival (PFS), disease‐specific survival (DSS), and recurrence‐free survival (RFS), as well as to determine the optimal NLR cut‐off value for distinguishing favorable and unfavorable prognosis groups in HNSCC patients.

## 2 Materials and Methods

### 2.1 Focused Question

The focused question “Does an elevated pretreatment NLR and increased TANs infiltration predict poorer survival outcomes in patients with HNSCC?” was formulated using the PICOS strategy:

POPULATION: Patients diagnosed with HNSCC confirmed by histopathology.

INTERVENTION: Measurement of pretreatment NLR in peripheral blood and assessment of TANs.

COMPARISON: Patients with lower NLR values below the cutoff threshold and lower TAN densities.

OUTCOMES: Survival endpoints including OS, PFS, DFS, DSS, and RFS expressed as hazard ratios (HR) with 95% confidence intervals (CI).

STUDY DESIGN: Human clinical studies, including cohort studies and retrospective analyses.

### 2.2 Strategies for Searching Relevant Scientific Articles

The Preferred Reporting Items for Systematic Reviews and Meta‐Analyses (PRISMA) guidelines have been applied in this systematic review to identify, evaluate, and explicate the strategies [22]. The study has been registered under the ID number CRD42023493614 at the Prospective Register of Systematic Reviews (Prospero). Using electronic databases, we conducted a literature search for scientific articles published before September 23, 2025, to investigate the association between HNSCC and NLR, as well as between HNSCC and TANs. The databases that we used were the Web of Science, Scopus, PubMed, and the Cochrane Library. The following Mesh terms were used: (“neutrophil‐lymphocyte ratio” OR “neutrophil‐to‐lymphocyte ratio” OR “neutrophil lymphocyte ratio” OR “tumour associated neutrophils” OR “intratumoural neutrophils” OR “tumor associated neutrophils” OR “intratumoral neutrophils”) AND (“pharynx cancer” OR “tongue cancer” OR “oropharynx cancer” OR “hypopharynx cancer” OR “buccal cancer” OR “mouth cancer” OR “larynx cancer” OR “oral cancer” OR “HNSCC” OR “head and neck squamous cell carcinoma”). The combination of characters has been modified to accommodate each search database.

### 2.3 Study Selection

**FIGURE 1:**
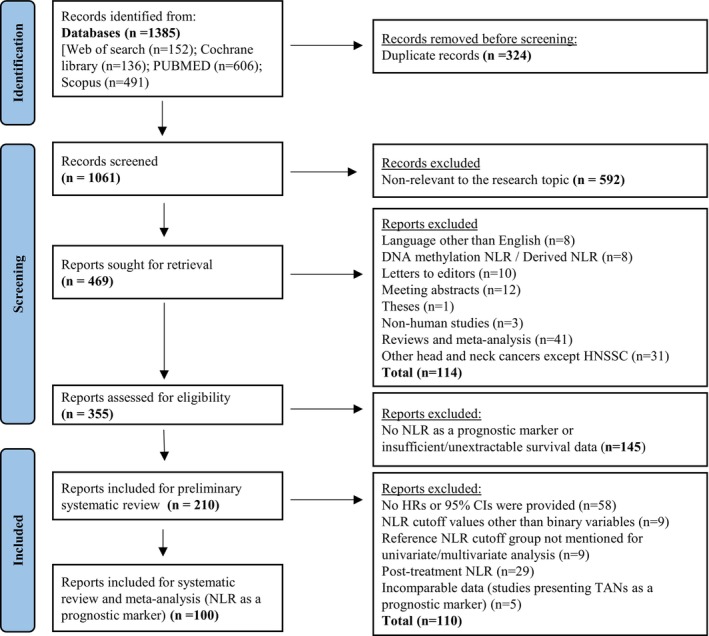
PRISMA flowchart showing the process of study screening for the systematic review and meta‐analysis.

The following criteria were prerequisites for inclusion in the systematic review: (1) studies assessing the prognostic value of pretreatment NLR in peripheral blood or TANs in HNSCC patients; (2) presentation of survival endpoints including OS, DFS, PFS, DSS, and RFS; (3) availability as a full‐text publication; and (4) accessibility in English. The exclusion criteria were as follows: (1) nonhuman studies; (2) studies in languages other than English; (3) studies focusing on cancers other than HNSCC; and (4) case studies, conference proceedings, correspondence or letters to editors, meta‐analyses, theses, or reviews. For the meta‐analysis, studies with incomplete statistical data, such as HR and 95% CI for the selected survival endpoints (OS/PFS/DSS/DFS/RFS), were excluded. Two authors (AKDVYR and AH) first reviewed publications by scanning the abstract and title. Next, AKDVYR and AH independently determined which articles to include and began retrieving each publication in preparation for the data extraction procedure. If disagreements arose about data elements, a third author (AAS) was consulted to resolve the discrepancies. The PRISMA flow chart for publication retrieval is shown in Figure 1.

### 2.4 Data Extraction

The following data were extracted from the included publications: name of the first author; year of publication; the country where the study was conducted; the number of patients (sample size); mean or median age of patients; follow‐up period; primary tumor site; tumor stage; cutoff methods and cutoff values for NLR; and HR, 95% CI, and *p*‐values for survival endpoints (OS, DFS, DSS, PFS, and RFS). The HRs, 95% CIs, and *p*‐values were extracted from both univariate and multivariate analyses.

### 2.5 Statistical Analysis

Meta‐analyses were performed using the “meta” package (version 4.8–1) in R (version 3.4.0). To determine the overall effect, compute pooled HRs, and evaluate the association between NLR and patients’ prognosis, a meta‐analysis was performed for study‐specific HR and 95% CI of OS, DFS, PFS, DSS, and RFS by generating forest plots using a random‐effects model [23]. Subgroup analyses were performed for tumor site and NLR cutoff value using HR data retrieved from the OS univariate analysis. Publication bias was evaluated using an Egger's regression intercept test and by visually analyzing the shifted funnel plot's asymmetry. Heterogeneity was evaluated using Cochrane's Q and Higgins’ I^2^ statistics. All statistical tests were considered statistically significant when the *p*‐value was less than 0.05.

### 2.6 Quality Control and Risk of Bias Assessment

The quality of the included studies for the meta‐analysis was independently assessed by two reviewers (AKDVYR and GVG) using the Reporting Recommendations for Tumor Marker Prognostic Studies (REMARK) guidelines. Six major criteria were evaluated: (1) patient samples, (2) clinical data, (3) NLR assessment, (4) prognostic data, (5) statistical analysis, and (6) classical prognostic factors (Table S1).

Risk of bias was assessed using the Meta‐Analysis of Statistics Assessment and Review Instrument (MASARIT), which includes 10 criteria: (Q1) inclusion criteria, (Q2) standardized NLR measurement, (Q3) NLR status identification, (Q4) consecutive inclusion, (Q5) complete participant inclusion, (Q6) demographic reporting, (Q7) clinical data reporting, (Q8) outcomes and follow‐up, (Q9) site/clinic demographics, and (Q10) statistical analysis appropriateness (Table S1). Question 4 was excluded due to inapplicability. If two authors disagreed on the scoring, a third author (AAS) was consulted, and a final decision was made. Scores were assigned to each category according to whether it was sufficient, insufficient, or not eligible for evaluation.

## 3 Results

### 3.1 Search Information

The initial search revealed 1385 articles. After removing duplicate articles (*n* = 324), 592 papers were excluded based on titles and abstracts that were irrelevant to the research topic. When applying the exclusion criteria, 114 articles were removed as follows: language other than English (*n* = 8), incomparable NLR measuring parameters such as DNA methylation NLR and derived NLR (*n* = 8), letters to editors (*n* = 10), meeting abstracts (*n* = 12), theses (*n* = 1), nonhuman studies (*n* = 3), reviews and meta‐analyses (*n* = 41), and studies on head and neck cancers other than HNSCC (*n* = 31). The remaining retrieved articles (*n* = 355) were reviewed in detail, leading to the removal of 145 articles that did not meet the inclusion criteria, as they either did not investigate NLR as a prognostic marker or did not report sufficient/extractable survival data. Consequently, 210 articles were evaluated for the preliminary systematic review. Out of these articles, an additional 110 were excluded because they did not report either HRs or 95% CI, or *p*‐values (*n* = 58), used NLR cutoff values other than binary variables (high and low NLR) (*n* = 9), lacked a reference NLR cutoff group for univariate/multivariate analysis (*n* = 9), reported post‐treatment NLR (*n* = 29) and presented TANs data that was incomparable with NLR data (*n* = 5). In total, 100 studies comprising 26 324 patients with HNSCC were selected for the final systematic review and meta‐analysis to assess NLR as a prognostic marker, as shown in the PRISMA flow chart (Figure 1). The main features of all selected studies for the systematic review and meta‐analysis regarding the prognosis of NLR [24, 25, 26, 27, 28, 29, 30, 31, 32, 33, 34, 35, 36, 37, 38, 39, 40, 41, 42, 43, 44, 45, 46, 47, 48, 49, 50, 51, 52, 53, 54, 55, 56, 57, 58, 59, 60, 61, 62, 63, 64, 65, 66, 67, 68, 69, 70, 71, 72, 73, 74, 75, 76, 77, 78, 79, 80, 81, 82, 83, 84, 85, 86, 87, 88, 89, 90, 91, 92, 93, 94, 95, 96, 97, 98, 99, 100, 101, 102, 103, 104, 105, 106, 107, 108, 109, 110, 111, 112, 113, 114, 115, 116, 117, 118, 119, 120, 121, 122, 123] and TANs [124, 125, 126, 127, 128] are summarized in Table S3, and Table S4, respectively.

### 3.2 Characteristics of the Eligible Studies Included in the Meta‐Analysis Evaluating the Prognostic Significance of NLR

All studies considered in the systematic review and meta‐analysis were published from 1990 to 2025, with the study population ranging from 41 to 1550 participants. These studies were carried out in 22 countries as listed in Table S3. Focusing on the most prominent survival endpoints, including OS, PFS, DFS, DSS, and RFS, this meta‐analysis was carried out using both univariate and multivariate statistical data separately. Furthermore, subgroup analysis for univariate OS was stratified by tumor site and NLR cut‐off tiers (1–1.99, 2–2.99, 3–3.99, 4–4.99, 5–5.99, and > 6). The 2–2.99 group was most common (40 studies), followed by 3–3.99 (26), 1–1.99 (14), 4–4.99 (11), 5–5.99 (6), and > 6 (3).

### 3.3 Quality and Risk Assessment

The amended REMARK and MASARIT criteria were used to assess each of the 100 studies included in the systematic review and meta‐analysis (Table S5). Based on both the REMARK and MASRIT criteria, all studies demonstrated a low risk of bias. The average quality score according to the REMARK criteria was 97.29%, while the MASARIT criteria yielded an average score of 98.22%.

### 3.4 Prognostic Impact of NLR on Survival Outcomes in HNSCC Patients

**FIGURE 2:**
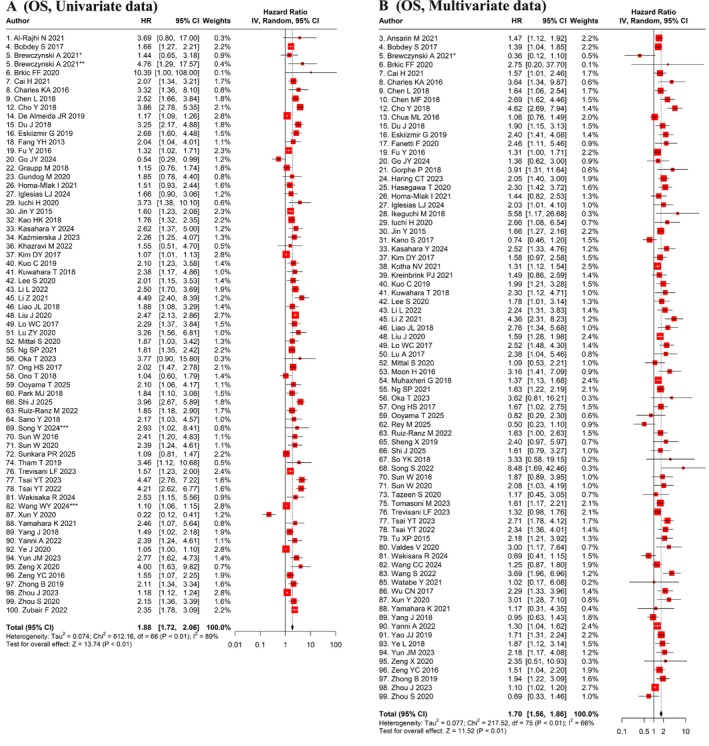
Meta‐analyses of the effect of NLR for Predicting Survival. Forest plot performed using the random effect statistical model using univariate (A) and multivariate data (B) for detecting prognostic efficacy of NLR on OS. CI, confidence interval; HR, hazard ratio; OS, overall survival. *HPV negative cohort; **HPV positive cohort; ***Training cohort.

**FIGURE 3:**
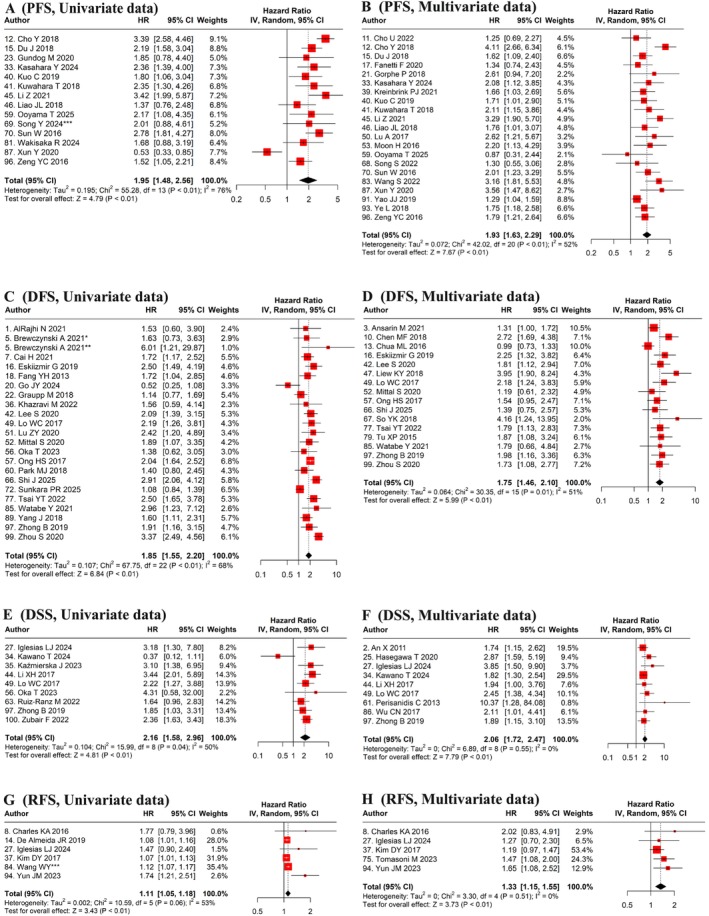
Meta‐analyses of the effect of NLR for Predicting Survival. Forest plot performed using the random effect statistical model using univariate and multivariate data for detecting prognostic efficacy of NLR on (A, B) PFS, (C, D) DFS, (E, F) DSS, and (G, H) RFS, respectively. CI, confidence interval; DFS, disease‐free survival; DSS, disease‐specific survival; HR, hazard ratio; RFS, recurrence‐free survival; PFS, progression‐free survival. *HPV negative cohort; **HPV positive cohort; ***Training cohort.

Univariate analyses of 67 cohorts, including 16 786 patients, revealed an HR for OS of 1.88 (95% CI: 1.72–2.06, *p* < 0.01), with significant heterogeneity (I^2^ = 89%, *p* < 0.01) (Table 1, Figure 2A). Multivariate analysis further supported this association, demonstrating a comparable pattern with considerable variability (HR: 1.70, 95% CI: 1.56–1.86, *p* < 0.01, I^2^ = 66%) across a larger patient cohort (76 studies, 20 576 patients; Table 1, Figure 2B). Similarly, PFS and DFS outcomes demonstrated a considerable association with NLR. The univariate analysis of PFS, comprising 14 studies with 2704 patients, revealed a hazard ratio of 1.95 (95% CI: 1.48–2.56, *p* < 0.01) with high heterogeneity (I^2^ = 76%, *p* < 0.01; Table 1, Figure 3A). Multivariate analysis of 21 studies, including 5764 patients, revealed a higher HR of 1.93 (95% CI: 1.63–2.29, *p* < 0.01) with moderate heterogeneity (I^2^ = 52%, p < 0.01; Table 1, Figure 3B). The univariate analysis of DFS comprising 23 studies, involving 4753 patients, showed a hazard ratio of 1.85 (95% CI: 1.55–2.20, *p* < 0.01) with moderately high heterogeneity (I^2^ = 68%, *p* < 0.01; Table 1, Figure 3C). The multivariate analysis of 16 cohorts (3836 patients) revealed a slightly reduced HR of 1.75 (95% CI: 1.46–2.10, p < 0.01) with moderate heterogeneity (I^2^ = 51%, *p* < 0.01; Table 1, Figure 3D).

**TABLE 1:** Key findings of the meta‐analysis for the impact of NLR on survival endpoints.

Survival endpoint	Statistical model	No. of cohorts/studies	No. of patients	HR (95% CI)	*p*	Heterogeneity test	
						*I* ^*2*^	*p*
OS	Univariate	67	16 786	1.88 (1.72–2.06)	< 0.01	89%	< 0.01
OS	Multivariate	76	20 576	1.70 (1.56–1.86)	< 0.01	66%	< 0.01
PFS	Univariate	14	2704	1.95 (1.48–2.56)	< 0.01	76%	< 0.01
PFS	Multivariate	21	5764	1.93 (1.63–2.29)	< 0.01	52%	< 0.01
DFS	Univariate	23	4753	1.85 (1.55–2.20)	< 0.01	68%	< 0.01
DFS	Multivariate	16	3836	1.75 (1.46–2.10)	< 0.01	51%	0.01
DSS	Univariate	9	2192	2.16 (1.58–2.96)	< 0.01	50%	0.04
DSS	Multivariate	9	1733	2.06 (1.72–2.47)	< 0.01	0%	0.55
RFS	Univariate	6	1643	1.11 (1.05–1.18)	< 0.01	53%	< 0.06
RFS	Multivariate	5	1453	1.33 (1.15–1.55)	< 0.01	0%	0.51

Results for DSS were particularly significant. Univariate analysis of 9 studies, involving 2192 patients, yielded an HR of 2.16 (95% CI: 1.58–2.96, *p* < 0.01; Table 1, Figure 3E). Multivariate analysis of 9 cohorts (1733 patients) strengthened this finding, showing an HR of 2.06 (95% CI: 1.72–2.47, *p* < 0.01; Table 1, Figure 3F). Notably, the heterogeneity was minimal and non‐significant in multivariate analyses (I^2^ = 0%, *p* = 0.55), whereas the univariate analysis showed moderate heterogeneity (I^2^ = 50%, *p* = 0.04). These findings highlight the strong prognostic significance of elevated NLR for DSS, indicating that patients with high NLR have more than twice the risk of disease‐specific mortality.

The HR reported in RFS studies was the lowest among all other survival endpoints, although it was slightly greater than 1. Univariate analysis of six studies (1643 patients) revealed an HR of 1.11 (95% CI: 1.05–1.18, *p* < 0.01) with non‐significant but moderate heterogeneity (I^2^ = 53%, *p* = 0.06), suggesting a weaker association (Table 1, Figure 3G). The multivariate analysis of five studies comprising 1453 patients demonstrated a similar pattern of risk associated with high NLR, with a pooled HR of 1.33 (95% CI: 1.15–1.55, *p* < 0.01). The heterogeneity was minimal and not statistically significant (I^2^ = 0%, *p* = 0.51; Table 1, Figure 3H). Collectively, these findings highlighted the prognostic relevance of NLR across different survival endpoints.

Evidence of publication bias was found in multiple analyses. The HR funnel plots for each of the selected survival endpoints (Figure S1) showed clear asymmetry, which suggests possible publication bias. This was further supported by Egger's regression test results, which found significant bias in several categories, including OS‐univariate (bias estimate = 2.60, *p* < 0.0001) and OS‐multivariate (bias estimate = 1.56, *p* < 0.0001). These results indicate a possible overrepresentation of studies with positive findings (Table S6).

### 3.5 Effect of NLR on OS Based on Tumor Location and NLR Cut‐Off Tiers

**FIGURE 4:**
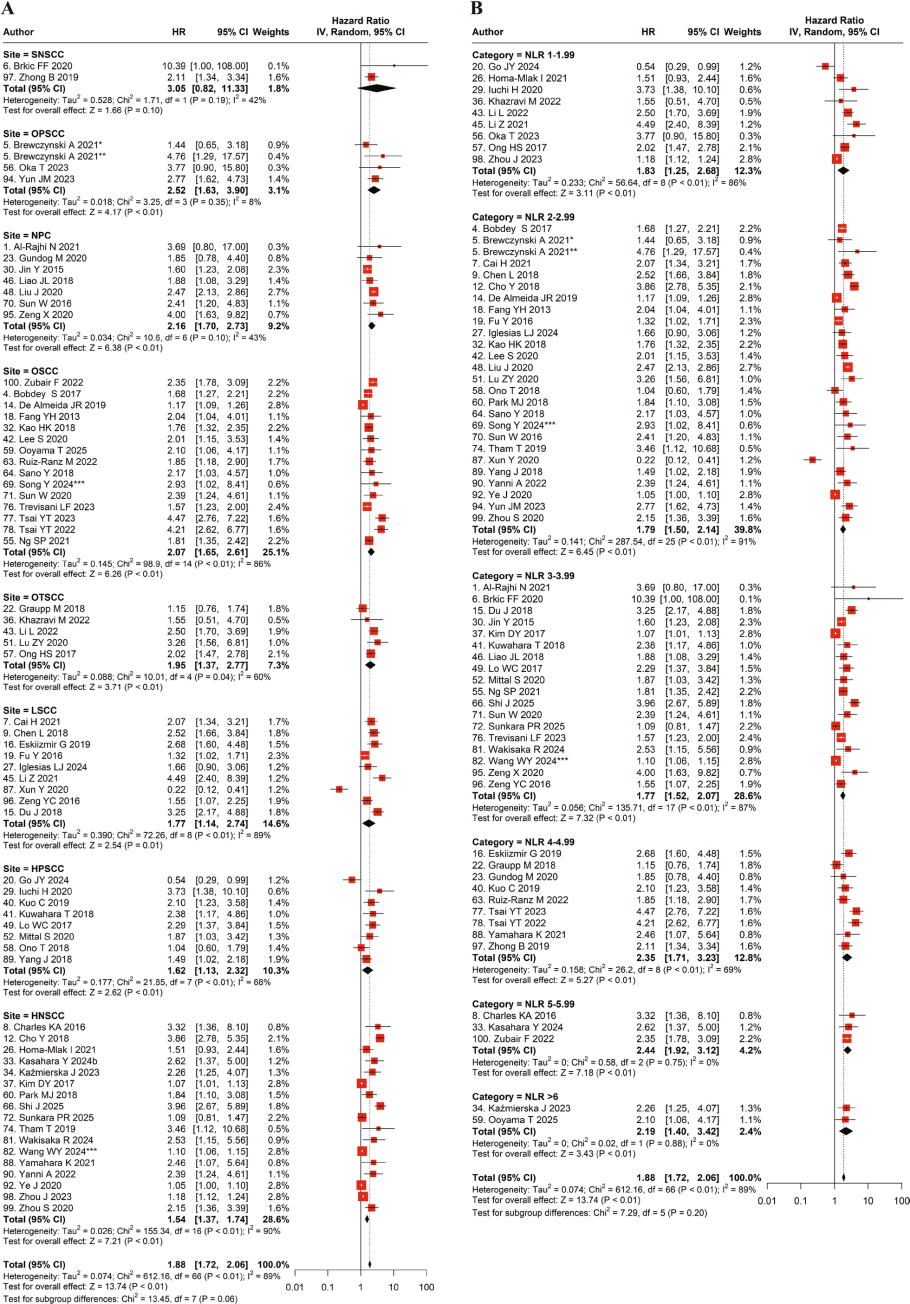
Meta‐analyses of the effect of tumor site and NLR cutoff value for predicting survival. Forest plot performed using the random effect statistical model using univariate overall survival data for detecting prognostic efficacy of NLR‐based on tumor site (A) and NLR cutoff point categorization (B). CI, confidence interval; HNSCC, head and neck squamous cell carcinoma; HPSCC, hypopharyngeal squamous cell carcinoma; HR, hazard ratio; LSCC, laryngeal squamous cell carcinoma; NLR, neutrophil‐to‐lymphocyte ratio; NPC, nasopharyngeal carcinoma; OPSCC, oropharyngeal squamous cell carcinoma; OSCC, oral squamous cell carcinoma; OTSCC, oral tongue squamous cell carcinoma; SNSCC, sinonasal squamous cell carcinoma. *HPV negative cohort; **HPV positive cohort; ***Training cohort.

Subgroup analysis, stratified using the univariate model on the OS endpoint, was conducted based on tumor location and NLR cutoff values (Figure 4, Table 2). High NLR was significantly associated with poor OS in the following tumor sites: oropharyngeal squamous cell carcinoma (OPSCC) HR = 2.52 (95% CI: 1.63–3.90, *p* < 0.01), nasopharyngeal carcinoma (NPC) HR = 2.16 (95% CI: 1.70–2.73, *p* < 0.01), oral squamous cell carcinoma (OSCC) HR = 2.07 (95% CI: 1.65–2.61, *p* < 0.01), oral tongue squamous carcinoma (OTSCC) HR = 1.95 (95% CI: 1.37–2.77, *p* < 0.01), laryngeal squamous carcinoma (LSCC) (HR = 1.77, 95% CI: 1.14–2.74, *p* = 0.01), hypopharyngeal squamous cell carcinoma (HPSCC) HR = 1.62 (95% CI: 1.13–2.32, *p* < 0.01), and HNSCC HR = 1.54 (95% CI: 1.37–1.74, *p* < 0.01; Figure 4A, Table 2). Sinonasal squamous cell carcinoma (SNSCC) was the only site in which high NLR showed no significant association with poor OS, HR = 3.05 (95% CI: 0.82–11.33, *p* = 0.1).

**TABLE 2:** The key findings of the subgroup analyses for the impact of NLR on overall survival.

Subgroup analysis/parameter	No. of cohorts/studies	No. of patients	HR (95% CI)	*p*	Heterogeneity test	
					*I* ^*2*^	*p*
Tumor location/type						
SNSCC	2	188	3.05 (0.82–11.33)	0.10	42%	0.19
OPSCC	4	712	2.52 (1.63–3.90)	< 0.01	8%	0.35
NPC	7	1554	2.16 (1.70–2.73)	< 0.01	43%	0.10
OSCC	15	5669	2.07 (1.65–2.61)	< 0.01	86%	< 0.01
OTSCC	5	749	1.95 (1.37–2.77)	< 0.01	60%	0.04
LSCC	9	2481	1.77 (1.14–2.74)	0.01	89%	< 0.01
HPSCC	8	915	1.62 (1.13–2.32)	< 0.01	68%	< 0.01
HNSCC	17	4518	1.54 (1.37–1.74)	< 0.01	90%	< 0.01
NLR cutoff point categorization						
1–1.99	9	1214	1.83 (1.25–2.68)	< 0.01	86%	< 0.01
2–2.99	26	6812	1.79 (1.50–2.14)	< 0.01	91%	< 0.01
3–3.99	18	5257	1.77 (1.52–2.07)	< 0.01	87%	< 0.01
4–4.99	9	1893	2.35 (1.71–3.23)	< 0.01	69%	< 0.01
5–5.99	3	1044	2.44 (1.92–3.12)	< 0.01	0%	0.75
> 6	2	359	2.19 (1.40–3.42)	< 0.01	0%	0.88
Overall NLR categorizations	67	16 786	1.88 (1.72–2.06)	< 0.01	89%	< 0.01

The heterogeneity levels within tumor locations are shown in Table 2. The highest heterogeneity was seen in HNSCC, LSCC, and OSCC. Moderate heterogeneity was observed in HPSCC, OTSCC, NPC, and SNSCC, whereas low heterogeneity was found in OPSSC. These findings indicate that tumor location significantly contributed to the observed heterogeneity.

In addition to the tumor location, NLR cutoff‐based subgroup analysis revealed a strong correlation between high NLR and low OS, as shown in Table 2. Higher HRs were observed in subgroups with NLR cut‐off values above 4 (HRs ranging from 2.19 to 2.44), compared to NLR cut‐off values below 4 (HRs ranging from 1.77 to 1.83), with the highest HR observed in the NLR 5–5.99 group with the poorest OS outcomes (HR = 2.44, 95% CI: 1.92–3.12, *p* < 0.01; Figure 4B). There was substantial variability in heterogeneity across the NLR cut‐off subgroups, with I^2^ values ranging from 0% to 91%. Based on the tumor location and NLR cutoff values, these subgroup analyses demonstrate the significant prognostic influence of NLR on OS in all subgroups. Therefore, in line with our results, an NLR cutoff value above 4 was associated with more than a two‐fold increase in HR, suggesting that this threshold may be appropriate for identifying HNSCC patients at higher risk.

### 3.6 Prognostic Significance of TANs in HNSCC Patients

In a systematic analysis of TANs, five articles revealed the prognostic significance in HNSCC patients. A German study analyzed a pooled cohort of 397 HNSCC patients from six different trials to assess OS based on the presence of CD66b^+^ TANs [126]. While individual cohort results were conflicting, the combined analysis found no significant association between CD66b^+^ TAN levels and OS [124]. A second article reported an investigation involving 81 patients with OSCC in China, where cancer‐specific survival (CSS) was assessed based on the density of CD15^+^ neutrophils, revealing an HR of 3.078 (95% CI: 1.196–7.922, *p* = 0.020) in multivariate analysis and highlighting the prognostic relevance of TANs [125]. Caldeira et al. conducted a preliminary study of 28 OSCC patients in a Brazilian cohort and discovered that advanced‐stage tumors (T3–T4) had significantly higher infiltration of CD66b^+^ TANs than early‐stage tumors (T1–T2) (*p* = 0.0002) [126]. Additionally, advanced tumor stages had a significantly higher CD66b/CD3 ratio (NLR) (*p* = 0.0006), suggesting a neutrophil‐dominant tumor microenvironment linked to tumor progression. Zhu et al. evaluated 61 patients with LSCC and reported that high intratumoral neutrophil density (CD66b^+^CD45^+^ cells > 50 per spot) was significantly correlated with poor OS in univariate analysis (HR = 4.637, 95% CI: 1.346–15.976, *p* = 0.015), although this association was not retained in multivariate analysis (HR = 2.949, 95% CI: 0.790–11.013, *p* = 0.108). Moreover, patients with a higher proportion of CXCR4^+^ TANs (> 20%) showed poorer OS (HR = 4.149, 95% CI: 1.076–15.995, *p* = 0.039), indicating that CXCR4^+^ neutrophils may serve as a prognostic indicator of poor outcome [127]. In a complementary study, Zhu et al. further evaluated 80 HNSCC patients to assess the prognostic role of TAN subtypes across different tumor regions. In the tumor nest, a higher N2/N1 TAN ratio was significantly associated with poorer OS (HR = 4.458, 95% CI: 1.384–14.366, *p* = 0.012), with similar findings in the tumor stroma (HR = 3.01, 95% CI: 1.072–8.45, *p* = 0.036) and combined tumor area (HR = 3.373, 95% CI: 1.269–8.965, *p* = 0.015) [128] (Table S4).

## 4 Discussion

This study is a comprehensive systematic review and meta‐analysis of 100 articles on the prognostic significance of NLR in HNSCC, as well as a review of five studies investigating the predictive role of TANs. The findings strongly support the prognostic significance of the NLR in HNSCC patients across multiple survival endpoints. Elevated NLR was consistently linked to worse OS, PFS, DFS, DSS, and RFS, with HRs ranging from 1.11 to 2.16. It is noteworthy that the highest reported HR was observed for DSS compared to other survival endpoints, which may be attributed to the specific method used to determine DSS.

The subgroup analysis indicates that the prognostic importance of NLR differs based on tumor location. For instance, HRs greater than 2 are notably observed in SNSCC, OPSSC, NPC, and OSCC. Previous survival meta‐analyses of the specified tumors in those locations have shown that patients with these tumors had poorer survival outcomes, indicating the aggressive nature of these tumors [129, 130, 131, 132, 133, 134]. Nevertheless, the observed HRs exceeding 1 across all HNSCC sites suggest that NLR remains a significant biomarker regardless of tumor location, despite some variability in the HR values between tumor sites.

Subgroup analysis utilizing NLR cutoff tiers demonstrated that its prognostic significance varies according to the selected cutoff point. Higher HRs were observed at elevated NLR cutoffs, particularly above 4, when compared to values below this threshold. Templeton et al. performed a meta‐analysis on various solid tumors and found that an NLR greater than 4 was associated with an HR of 1.81 for OS, reinforcing the broader prognostic importance of this threshold [19]. Overall, our findings indicate that an NLR value above 4 could be a reliable prognostic marker for patients with HNSCC, as evidenced by the reported over 2‐fold higher HR in this meta‐analysis.

With 100 articles including 26 324 HNSCC patients, this review and meta‐analysis is the most extensive to date. Nevertheless, some limitations must be acknowledged. Studies generally exhibited high methodological quality, with REMARK and MASARIT scores exceeding 97%, indicating low bias risk. However, funnel plot asymmetry suggests possible publication bias, likely related to the retrospective nature of most included studies and selective reporting. The observed disparity suggests that even though many studies may be well‐reported and methodologically well arranged, studies with positive or statistically significant results may be more likely to be published than negative results. Also, smaller studies with bigger effects are more likely to be published, which may cause funnel plot asymmetry even when REMARK and MASARIT scores are high. Our inclusion criteria, focusing on studies reporting dichotomized NLR cutoffs and hazard ratios, may also have introduced selective reporting bias.

Our analyses of survival endpoints reveal a heterogeneity that spans from the lowest (0%) to the highest (89%). Several factors may contribute to the observed significant heterogeneity (I^2^), including a predominance of data from Asian populations, varying NLR cutoff values and methods, different approaches of HR analysis, and varying disease stages and tumor sites. The significant variation seen in certain analyses emphasizes the need for careful interpretation as well as the value of subgroup analyses in identifying the underlying causes, as these elements may also result in potential publication bias.

Overall, this systematic review's findings emphasize TANs' importance as tumor‐specific inflammatory markers and the practicality of using TANs for high‐risk patient identification. High levels of TANs, especially CD15^+^ and CXCR4^+^ subsets, along with a higher N2/N1 TAN ratio in the tumor nest and stroma, have been consistently associated with poor survival in many HNSCC subtypes; however, their impact may vary depending on tumor stage and TANs' location [124, 125, 126, 127, 128]. The link between poor prognosis and increased NLR may result from the higher presence of TANs in the tumor microenvironment, which could encourage tumor progression and metastasis.

Overall, the findings of this study offer insightful information about the prognostic significance of NLR and TANs in patients with HNSCC. It also underscores the importance of accounting for tumor site and cutoff value when interpreting NLR‐based prognosis.

## 5 Conclusion

This systematic review and meta‐analysis suggest that an NLR cutoff value above 4 may serve as a reliable prognostic marker in HNSCC patients. However, further prospective studies are needed to validate its clinical utility and support its integration into routine practice.

## Author Contributions

A.K.D.V.Y.R., A.H., and A.A.‐S. performed study concept and design. A.K.D.V.Y.R., G.V.G., A.H., and A.A.‐S. performed the development of the methodology and reviewed the data. A.K.D.V.Y.R. and M.T. performed statistical analysis. A.K.D.V.Y.R., A.H., and A.A.‐S. provided interpretation of data. A.K.D.V.Y.R. wrote the manuscript. All authors have read, revised, and approved the final paper.

## Funding

This work is funded by the Research Council of Finland, the Sigrid Jusélius Foundation, the Minerva Foundation, and the Finnish Dental Society Apollonia.

## Conflicts of Interest

The authors declare no conflicts of interest.

## Supporting information


**TABLE S1:** REMARK (Reporting Recommendations for Tumor Marker Prognostic Studies) criteria for quality Analysis to assess NLR as a prognostic marker in HNSCC.
**TABLE S2:** Meta‐Analysis of Statistics Assessment and Review Instrument (MASARATI) for evaluating NLR as a Prognostic Marker in HNSCC.
**TABLE S3:** Studies included in the systematic review and meta‐analysis for evaluating the prognostic significance of NLR in HNSCC patients.
**TABLE S4:** Included studies in the systematic review for evaluating the prognostic significance of tumor associated neutrophils (TANs) in HNSCC patients.
**TABLE S5:** Quality Analysis for NLR as a Prognostic Marker in HNSCC based on REMARK (C1‐C6) and MASARATI Criteria (Q1‐Q10).
**TABLE S6:** Results of Egger's regression test for assessing publication bias in included studies.
**FIGURE S1:** Funnel plots of studies included in the meta‐analysis evaluating the association between neutrophil‐to‐lymphocyte ratio (NLR) and survival outcomes, used to assess potential publication bias. Plots correspond to: A—Overall survival (OS), univariate data; B—OS, multivariate data; C—Progression‐free survival (PFS), univariate data; D—PFS, multivariate data; E—Disease‐free survival (DFS), univariate data; F—DFS, multivariate data; G—Disease‐specific survival (DSS), univariate data; H—DSS, multivariate data; I—Recurrence‐free survival (RFS), univariate data; J—RFS, multivariate data. The horizontal axis and the vertical axis represent the hazard ratio (HR) and the standard error of the effect size, respectively.

## Data Availability

Data sharing not applicable to this article as no datasets were generated or analysed during the current study.
