# Supplementary material for: Prognostic Significance of the Pretreatment Neutrophil‐to‐Lymphocyte Ratio and Tumor‐Associated Neutrophils in Head and Neck Squamous Cell Carcinoma: Systematic Review and Meta‐Analysis
Source: J Oral Pathol Med. 2025 Dec 30;55(4):458–72. doi: 10.1111/jop.70110 (PMC13065941; doi:10.1111/jop.70110)
Supplement: Supplementary file 1 — TABLE S1: REMARK (Reporting Recommendations for Tumor Marker Prognostic Studies) criteria for quality Analysis to assess NLR as a prognostic marker in HNSCC. TABLE S2: Meta‐Analysis of Statistics Assessment and Review Instrument (MASARATI) for evaluating NLR as a Prognostic Marker in HNSCC. TABLE S3: Studies included in the systematic review and meta‐analysis for evaluating the prognostic significance of NLR in HNSCC patients. TABLE S4: Included studies in the systematic review for evaluating the prognostic significance of tumor associated neutrophils (TANs) in HNSCC patients. TABLE S5: Quality Analysis for NLR as a Prognostic Marker in HNSCC based on REMARK (C1‐C6) and MASARATI Criteria (Q1‐Q10). TABLE S6: Results of Egger's regression test for assessing publication bias in included studies. FIGURE S1: Funnel plots of studies included in the meta‐analysis evaluating the association between neutrophil‐to‐lymphocyte ratio (NLR) and survival outcomes, used to assess potential publication bias. Plots correspond to: A—Overall survival (OS), univariate data; B—OS, multivariate data; C—Progression‐free survival (PFS), univariate data; D—PFS, multivariate data; E—Disease‐free survival (DFS), univariate data; F—DFS, multivariate data; G—Disease‐specific survival (DSS), univariate data; H—DSS, multivariate data; I—Recurrence‐free survival (RFS), univariate data; J—RFS, multivariate data. The horizontal axis and the vertical axis represent the hazard ratio (HR) and the standard error of the effect size, respectively. [file JOP-55-458-s001.docx]

**Supplementing information**

**TABLE S1. REMARK (Reporting Recommendations for Tumor Marker Prognostic Studies) criteria for quality Analysis to assess NLR as a prognostic marker in HNSCC**

| **Checklist items** | **Criteria** |
| --- | --- |
| **C1) Patient samples** | The study cohort is well-defined, and necessary information about its population is provided, such as the number of studied patients, source of the sample, study period, and follow-up time. |
| **C2) Clinical data of the cohort** | The basic clinical data, including age, gender, and clinical stage of cancer/TNM, were provided. If applicable, other relevant factors such as HPV status and comorbidities were reported. |
| **C3) NLR assessment** | The method for measuring NLR and the time point of measurement (pre-treatment, post-treatment, or during follow-up) were defined. The study reported NLR values as a continuous measure or categorized into high vs. low groups, with the cutoff value specified either in advance or later in the analysis. |
| **C4) Prognostics** | The endpoints of survival analyses were clearly defined (e.g., overall survival (OS), disease-free survival (DFS), disease-specific survival (DSS), progression-free survival (PFS), recurrence-free survival (RFS)). Follow-up duration and how patients were counted in survival analysis, including handling missing data and patients lost to follow-up, were explained. |
| **C5) Statistics** | Estimated effects (e.g., hazard ratio (HR), confidence intervals (CI)) describing the relationship between NLR status (high vs. low) and clinical outcomes (e.g., OS, PFS) were provided. Adequate statistical analyses (e.g., Cox regression modeling) were performed to adjust for known prognostic factors such as age, stage, etc. |
| **C6) Classical prognostic factors** | The prognostic value of classical prognostic factors was reported. The relationship between the evaluated NLR status and classical prognostic factors in predicting HNSCC progression was discussed. |

**TABLE S2. Meta-Analysis of Statistics Assessment and Review Instrument (MASARATI) for evaluating NLR as a Prognostic Marker in HNSCC**

| **Checklist Items** | **Questions** | **Criteria** |
| --- | --- | --- |
| **Q1. Criteria for inclusion** | Were there clear criteria for inclusion in the case series? | The studies clearly defined inclusion criteria, specifying the population (HNSCC patients), the assessment of NLR (Neutrophil-to-Lymphocyte Ratio), and the outcome measures (e.g., overall survival, progression-free survival). |
| **Q2. Standardized measurement of NLR** | Was NLR measured in a standard, reliable way for all participants included in the studies? | The method for measuring NLR was consistent across studies, including time point of measurement (pre-treatment, post-treatment, follow-up), and the technique for calculating the NLR was standardized. |
| **Q3. Identification of NLR status** | Were valid methods used for identifying the NLR status (high vs. low) for all participants included in the studies? | The method for classifying patients into high or low NLR groups was clearly defined, and the cut-off values used to distinguish between high and low NLR were either pre-specified or explained in the study. |
| **Q4. Consecutive inclusion of participants** | Did the case series have consecutive inclusion of participants? | Not applicable (N/A) for meta-analysis. |
| **Q5. Complete inclusion of participants** | Did the case series have a complete inclusion of participants? | All eligible participants who met the inclusion criteria were included in the study. The studies clearly reported any exclusions or lost follow-up, if applicable. |
| **Q6. Reporting of participant demographics** | Was there clear reporting of the demographics of the participants in the study? | Demographic information, including age, gender, and clinical characteristics of participants, was clearly reported. The studies also included information on important confounders such as HPV status, which could affect NLR. |
| **Q7. Reporting of clinical information** | Was there clear reporting of clinical information of the participants? | The clinical information (e.g., tumor stage, treatment modality, co-morbidities) was adequately reported. |
| **Q8. Reporting of outcomes and follow-up results** | Were the outcomes or follow-up results of cases clearly reported? | The survival outcomes (e.g., overall survival, progression-free survival) and follow-up results for patients were reported. Any missing data or loss to follow-up was properly addressed. |
| **Q9. Reporting of site/clinic demographic information** | Was there clear reporting of the presenting site(s)/clinic(s) demographic information? | Demographic information regarding the participating sites/clinics was provided to ensure the generalizability of the study results. |
| **Q10. Appropriateness of statistical analysis** | Was statistical analysis appropriate? | Statistical analysis (e.g., Cox regression models, Kaplan-Meier survival curves, hazard ratios) was appropriately used to evaluate the prognostic significance of NLR, with adjustment for confounders. |

**TABLE S3. Studies included in the systematic review and meta-analysis for evaluating the prognostic significance of NLR in HNSCC patients**

| **No.** | **First author and reference number** | **Year** | **Country** | **Tumor site** | **Study period / Median (IQR)** | **No. cases** | **HPV/ EBV** | **Size** | **NLR cut-off method** | **NLR cut-off value** | **Survival endpoint** | **HR (95 % CI)** | **P** |
| --- | --- | --- | --- | --- | --- | --- | --- | --- | --- | --- | --- | --- | --- |
| 1 | Al-Rajhi N ^24^ | 2021 | Saudi Arabia | NPC | Med - 4.7 years | 83 |  | T1-4, III-IV, N0-N3 | Literature | 3 | OS uni | 7.6 (0.8–17.0) | 0.083 |
|  |  |  |  |  |  |  |  |  |  |  | OS multi |  | 0.908 |
|  |  |  |  |  |  |  |  |  |  |  | DFS uni | 1.6 (0.6–3.9) | 0.341 |
|  |  |  |  |  |  |  |  |  |  |  | DFS multi |  | 0.446 |
| 2 | An X ^25^ | 2011 | China | NPC | 11/2001–07/2009 | 363 |  | I-IV | Receiver Operating Characteristic (ROC) curve | 3.73 | DSS multi | 1.74 (1.15–2.62) | 0.008 |
|  |  |  |  |  |  |  |  |  |  |  | DMFS multi | 2.37 (1.37–4.10) | 0.002 |
| 3 | Ansarin M ^26^ | 2021 | Italy | OTSCC | 01/2000–02/2020, Med- 5.01 years (0–18.68) | 577 |  | T1-4, I-IV, N0-N+ | Literature | 3 | OS multi | 1.47 (1.12–1.92) | 0.004 |
|  |  |  |  |  |  |  |  |  |  |  | DFS multi | 1.31 (1.00–1.72) | 0.04 |
|  |  |  |  |  |  |  |  |  |  |  | CSS multi | 1.43 (1.04–1.98) | 0.03 |
| 4 | Bobdey S ^27^ | 2017 | India | OSCC | 01/ 2007–12/2008 | 471 |  | T1-4, I-IV, N0-N+ | ROC curve | 2.38 | OS uni | 1.676 (1.271–2.209) | 0.000 |
|  |  |  |  |  |  |  |  |  |  |  | OS multi | 1.392 (1.045–1.855) | 0.024 |
| 5 | Brewczynski A ^28^ | 2021 | Poland | OPSCC | Med-74.58 months (0.1–165.58) | 127 | HPV + * (59) / HPV- ** (68) | T1-4, I-III, N0-N3 | ROC curve | OS - 2.13, DFS- 2.29 | OS uni (HPV-*) | 1.44 (0.65–3.18) | 0.364 |
|  |  |  |  |  |  |  |  |  |  |  | OS multi (HPV-*) | 0.36 (0.12–1.10) | 0.074 |
|  |  |  |  |  |  |  |  |  |  |  | OS uni (HPV+**) | 4.76 (1.29–17.57) | 0.019 |
|  |  |  |  |  |  |  |  |  |  |  | DFS uni (HPV-**) | 1.63 (0.73–3.63) | 0.232 |
|  |  |  |  |  |  |  |  |  |  |  | DFS uni (HPV+) | 6.02 (1.21–29.87) | 0.028 |
| 6 | Brkic FF ^29^ | 2020 | Austria | SNSCC | 2002–2015 | 41 |  | T1-4, I-III, N0-N+ | Median value | 3.5 | OS uni | 3.36 (1.0–108) | 0.041 |
|  |  |  |  |  |  |  |  |  |  |  | OS multi | 2.99 (0.2–37.7) | 0.396 |
| 7 | Cai H ^30^ | 2021 | China | LSCC | 12/2008–09/2014 | 203 |  | T1-4, N0-N+ | ROC curve | 2.41 | OS uni | 2.074 (1.340 3.209) | 0.001 |
|  |  |  |  |  |  |  |  |  |  |  | OS multi | 1.573 (1.006–2.459) | 0.047 |
|  |  |  |  |  |  |  |  |  |  |  | DFS uni | 1.716 (1.170–2.518) | 0 .006 |
| 8 | Charles KA ^31^ | 2016 | Australia | ^HNSCC^ | 01/2005–01/2012 | 145 |  | T1-4, I-IV, N0-N3 | Literature | 5 | OS uni | 3.32 (1.36–8.10) | <0.01 |
|  |  |  |  |  |  |  |  |  |  |  | OS multi | 3.64 (1.34–9.87) | 0.02 |
|  |  |  |  |  |  |  |  |  |  |  | RFS uni | 1.76 (0.79–3.96) | 0.2 |
|  |  |  |  |  |  |  |  |  |  |  | RFS multi | 2.02 (0.83–4.91) | 0.1 |
| 9 | Chen L ^32^ | 2018 | China | LSCC | 01/2010–12/2017, Med- 47 months (4–98) | 361 |  | T1-4, I-IV, N0-N2 | ROC curve | 2.45 | OS uni | 2.53 (1.66–3.84) | 0.001 |
|  |  |  |  |  |  |  |  |  |  |  | OS multi | 1.64 (1.06–2.54) | 0.026 |
| 10 | Chen MF ^33^ | 2018 | Taiwan | HNSCC | Med- 25.6 (1.37–148) months | 227 |  | I-IV, N0-N+ | ROC curve | 3 | OS multi | 2.69 (1.62–4.46) | 0.001 |
|  |  |  |  |  |  |  |  |  |  |  | DFS multi | 2.71 (1.69–4.38) | 0.001 |
| 11 | Cho U ^34^ | 2022 | Korea | OSCC | 01/2003–12/2019 | 269 |  | T1-4, I-IV, N0-N4 | ROC curve | 1.7584 | DSS uni |  | 0.122 |
|  |  |  |  |  |  |  |  |  |  |  | DSS multi |  | N/A |
|  |  |  |  |  |  |  |  |  |  |  | PFS uni |  | 0.048 |
|  |  |  |  |  |  |  |  |  |  |  | PFS multi | 1.25 (0.69–2.27) | 0.455 |
| 12 | Cho Y ^35^ | 2018 | Korea | HNSCC | 2006–2015, Med- 39 months (2–130) | 621 |  | T1-4, I-IV, N0-N3 | ROC curve | 2.7 | OS uni | 3.86 (2.78–5.35) | <0.001 |
|  |  |  |  |  |  |  |  |  |  |  | OS multi | 4.63 (2.69–7.94) | <0.001 |
|  |  |  |  |  |  |  |  |  |  |  | PFS uni | 3.39 (2.58–4.46) | 0.001 |
|  |  |  |  |  |  |  |  |  |  |  | PFS multi | 4.10 (2.66–6.34) | 0.001 |
| 13 | Chua ML ^36^ | 2016 | Singapore | NPC | 09/1997–05/2003 | 380 | EBV +/- | T1-4, I-IV, N0-N3 | Median value and literature | 3.0 | OS multi | 1.06 (0.76–1.49) | 0.7 |
|  |  |  |  |  |  |  |  |  |  |  | DFS multi | 0.98 (0.73–1.33) | 0.9 |
|  |  |  |  |  |  |  |  |  |  |  | DMFS multi | 1.46 (0.89–2.41) | 0.1 |
|  |  |  |  |  |  |  |  |  |  |  | LRFS multi | 1.55 (0.90–2.67) | 0.1 |
| 14 | De Almeida JR ^37^ | 2019 | Canada | OSCC | 1998–2011 | 551 |  | T1-4, N0-N+ | Contal and O’Quigley method | 2.9 | OS uni | 1.17 (1.09–1.26) | < 0.001 |
|  |  |  |  |  |  |  |  |  |  |  | OS uni | 1.05 (1.02–1.09) | 0.004 |
|  |  |  |  |  |  |  |  |  |  |  | RFS uni | 1.08 (1.01–1.16) | 0.03 |
| 15 | Du J ^38^ | 2018 | China | LSCC | 09/2008-09/2013 | 654 |  | T1-4, I-IV, N0-N3 | Classification and regression tree (CART) | 3.18 | OS uni | 3.254 (2.171–4.877) | <0.0001 |
|  |  |  |  |  |  |  |  |  |  |  | OS multi | 1.901 (1.153–3.135) | 0.012 |
|  |  |  |  |  |  |  |  |  |  |  | PFS uni | 2.191 (1.582–3.035) | <0.001 |
|  |  |  |  |  |  |  |  |  |  |  | PFS multi | 1.621 (1.094–2.404) | 0.016 |
| 16 | Eskiizmir G ^39^ | 2019 | Turkey | LSCC | 2002–2015 | 229 |  | T1-4, I-IV, N0-N2 | Literature | 4 | OS uni | 2.675 (1.597–4.483) | <0.001 |
|  |  |  |  |  |  |  |  |  |  |  | OS multi | 2.396 (1.408–4.077) | 0.001 |
|  |  |  |  |  |  |  |  |  |  |  | DFS uni | 2.500 (1.492–4.189) | 0.001 |
|  |  |  |  |  |  |  |  |  |  |  | DFS multi | 2.246 (1.322–3.816) | 0.006 |
|  |  |  |  |  |  |  |  |  |  |  | LRFS uni | 2.498 (1.491–4.185) | 0.001 |
|  |  |  |  |  |  |  |  |  |  |  | LRFS multi | 2.210 (1.301–3.753) | 0.003 |
| 17 | Fanetti F ^40^ | 2020 | Italy | OPSCC | 01/2010–12/2015,  Med-50 months (5–95) | 125 | HPV +/- | I-IV | Median | 3 | OS multi | 2.46 (1.11–5.46) | 0.03 |
|  |  |  |  |  |  |  |  |  |  |  | PFS multi | 1.34 (0.74–2.43) | 0.33 |
| 18 | Fang YH ^41^ | 2013 | Taiwan | OSCC | 07/2007–04/2012 | 226 |  | T1-4, I-IV, N0-N3 | Median value | 2.44 | OS uni | 2.04 (1.036–4.014) | 0.034 |
|  |  |  |  |  |  |  |  |  |  |  | DFS uni | 1.72 (1.038–2.849) | 0.031 |
| 19 | Fu Y ^42^ | 2016 | China | LSCC | 01/1990–07/2010,  Med - 62.28 months | 420 |  | T3-4, III-IV, N0-N3 | ROC curve | 2.59 | OS uni | 1.32 (1.02–1.71) | 0.032 |
|  |  |  |  |  |  |  |  |  |  |  | OS multi | 1.31 (1.00–1.71) | 0.046 |
|  |  |  |  |  |  |  |  |  |  |  | CSS uni | 1.42 (1.07–1.88) | 0.015 |
|  |  |  |  |  |  |  |  |  |  |  | CSS multi | 1.42 (1.06–1.91) | 0.018 |
| 20 | Go JY ^43^ | 2024 | Korea | HPSSC | 01/2009–12/2019 | 101 |  | T1-4, I-IV, N0-N3 | Median | 1.92 | OS (uni) | 0.54 (0.29–0.99) | 0.047 |
|  |  |  |  |  |  |  |  |  |  |  | OS (multi) | 1.36 (0.62–3.00) | 0.447 |
|  |  |  |  |  |  |  |  |  |  |  | DFS (uni) | 0.52 (0.25–1.08) | 0.081 |
| 21 | Gorphe P ^44^ | 2018 | France | OPSCC | 2003–2016,  Med – 32 months | 167 | HPV +/- | I-III | Literature (5) &  Median value (2.65) | 5 and 2.65 | OS uni > 5 |  | 0.001 |
|  |  |  |  |  |  |  |  |  |  |  | OS multi >5 | 3.910 (1.313–11.643) | 0.014 |
|  |  |  |  |  |  |  |  |  |  |  | PFS uni >5 |  | 0.015 |
|  |  |  |  |  |  |  |  |  |  |  | PFS multi >5 | 2.609 (0.945–7.204) | 0.064 |
|  |  |  |  |  |  |  |  |  |  |  | OS uni >2.65 |  | 0.227 |
|  |  |  |  |  |  |  |  |  |  |  | OS multi >2.65 |  | No |
|  |  |  |  |  |  |  |  |  |  |  | PFS uni >2.65 |  | 0.190 |
|  |  |  |  |  |  |  |  |  |  |  | PFS multi >2.65 |  | No |
| 22 | Graupp M ^45^ | 2018 | Austria | OTSSC | 01/2002–06/2015 | 197 |  | T1-4, I-IV, N0-N3 | Median value | 4 | OS uni | 1.147 (0.759–1.735) | 0.515 |
|  |  |  |  |  |  |  |  |  |  |  | DFS uni | 1.140 (0.767–1.694) | 0.517 |
| 23 | Gundog M ^46^ | 2020 | Turkey | NPC | 2010–2018 | 97 |  | T1-4, II-IV, N0-N3 | ROC curve | 4.42 | OS uni | 1.84 (0.775–4.400) | 0.16 |
|  |  |  |  |  |  |  |  |  |  |  | OS multi |  | No |
|  |  |  |  |  |  |  |  |  |  |  | PFS uni | 1.84 (0.775–4.400) | 0.16 |
|  |  |  |  |  |  |  |  |  |  |  | PFS multi |  | No |
| 24 | Haring CT ^47^ | 2023 | United States | HNSCC | 1998–2019,  Mean -14.4 months (SD, 15.9) | 447 | HPV +/- | I-IV | Mean value | 10 | OS multi | 2.1 (1.4–3.0) | <0.001 |
| 25 | Hasegawa T ^48^ | 2020 | Japan | OSCC | 01/2001–12/2013,  Med - 59.1 months (1–179) | 433 |  | T1-4, N0-N+ | ROC curve | 2.22 | OS multi | 2.30 (1.42–3.72) | <0.001 |
|  |  |  |  |  |  |  |  |  |  |  | DSS multi | 2.87 (1.59–5.19) | <0.001 |
| 26 | Homa-Mlak I ^49^ | 2021 | Poland | HNSCC | 2014–2017,  Med -27 months | 207 |  | III-IV |  | 1.76 | OS uni | 1 (0.93–2.44) | 0.1443 |
|  |  |  |  |  |  |  |  |  |  |  | OS multi | 1.44 (0.82–2.53) | 0.2074 |
| 27 | Iglesias LJ ^50^ | 2024 | Spain | LSCC | 1998–2019,  Med -54.6 months (24–195) | 201 |  | T1-2, I-II | ROC curve | 2.44 | OS uni | 1.6 (0.9–3.06) | 0.11 |
|  |  |  |  |  |  |  |  |  |  |  | DSS uni | 3.23 (1.3–7.8) | 0.009 |
|  |  |  |  |  |  |  |  |  |  |  | RFS uni | 1.45 (0.9–2.4) | 0.15 |
|  |  |  |  |  |  |  |  |  |  |  | OS multi | 2.05 (1.01–4.1) | 0.047 |
|  |  |  |  |  |  |  |  |  |  |  | DSS multi | 3.8 (1.5–9.9) | 0.006 |
|  |  |  |  |  |  |  |  |  |  |  | RFS multi | 1.3 (0.7–2.3) | 0.37 |
| 28 | Ikeguchi M ^51^ | 2016 | Japan | HPSCC | 2002007–12 Mean - 38.5 months (5–108) | 59 |  | III-IV |  | 5 | OS uni |  | <0.001 |
|  |  |  |  |  |  |  |  |  |  |  | OS multi | 5.586 (1.169–26.68) | 0.031 |
| 29 | Iuchi H ^52^ | 2020 | Japan | HPSCC | 01/2007–12/2017,  Med- 61 months (1–127) | 106 |  | T2-4, II-IV, N0-N3 | ROC curve | 1.8 | OS uni (all patients) | 3.73 (1.38–10.10) | 0.0096 |
|  |  |  |  |  |  |  |  |  |  |  | OS multi (all patients) | 2.66 (1.08–6.54) | 0.034 |
|  |  |  |  |  |  |  |  |  |  |  | OS uni (stage IV patients) | 0.36 (0.12–1.08) | 0.069 |
|  |  |  |  |  |  |  |  |  |  |  | OS multi (stage IV patients) |  | >0.05 |
| 30 | Jin Y ^53^ | 2015 | China | NPC | 01/2006–12/2012 | 229 |  | N0-N+ | Median value | 3.6 | OS uni | 1.604 (1.234–2.084) | < .001 |
|  |  |  |  |  |  |  |  |  |  |  | OS multi | 1.662 (1.275–2.165) | < .001 |
| 31 | Kano S ^54^ | 2017 | Japan | HNSCC | 01/2003–12/2012,  Med- 5.1 years (0.3–12) | 285 |  | T1-4, I-IV, N0-N3 | ROC curve | 1.92 | OS multi | 0.742 (0.458–1.204) | 0.228 |
| 32 | Kao HK ^55^ | 2018 | Taiwan | OSCC | 09/2005–12/2014 | 613 |  | T1-4, I-IV, N0-N2 | Median value | 2.28 | OS uni | 1.759 (1.320–2.345) | 0.0001 |
| 33 | Kasahara Y ^56^ | 2024b | Japan | HNSCC | 04/2020–03/2023 | 74 | p16+ - 7/74 |  | Previous report | 5 | OS (uni) | 2.63 (1.37–5.00) | 0.0036 |
|  |  |  |  |  |  |  |  |  |  |  | OS (multi) | 2.56 (1.33–4.76) | 0.0049 |
|  |  |  |  |  |  |  |  |  |  |  | PFS (uni) | 2.38 (1.39–4.00) | 0.0015 |
|  |  |  |  |  |  |  |  |  |  |  | PFS (multi) | 2.08 (1.12–3.85) | 0.0216 |
| 34 | Kawano T ^57^ | 2024 | Japan | LSCC | 01/2010–12/2016 | 125 |  | T1-5, I-IV, N- N+ | ROC | 1.88 | DSS (uni) | 0.365 (0.120–1.112) | 0.0762 |
|  |  |  |  |  |  |  |  |  |  |  | DSS (multi) | 1.819 (1.302–2.542) | 0.0005 |
| 35 | Kaźmierska J ^58^ | 2023 | Poland | HNSCC | 01/2010–01/2023,  Med-27.8 months (0–139) | 317 | HPV +/- | T1-4, I-IV, N0-N3 |  | 1 log NLR | OS uni | 2.26 (1.25–4.07) | 0.0068 |
|  |  |  |  |  |  |  |  |  |  |  | OS multi | 1.93 (1.22–3.1) | 0.005 |
|  |  |  |  |  |  |  |  |  |  |  | DSS uni | 3.1 (1.38–6.95) | 0.006 |
| 36 | Khazravi M ^59^ | 2022 | Iran | OTSCC | 2011–2018 | 129 |  | T1-4, N0-N+ | ROC curve | 1.21 | OS uni | 1.51 (0.51–4.70) | 0.43 |
|  |  |  |  |  |  |  |  |  |  |  | DFS uni | 1.56 (0.59–4.14) | 0.36 |
| 37 | Kim DY ^60^ | 2017 | Korea | HNSCC | 01/2009–02/2015,  Med- 39 (10–62) months | 104 |  | T1-4, III-IV, N0-N3 | ROC curve | 3 | OS uni | 1.07 (1.01–1.13) | 0.003 |
|  |  |  |  |  |  |  |  |  |  |  | OS multi | 1.52 (0.97–2.58) | 0.156 |
|  |  |  |  |  |  |  |  |  |  |  | RFS uni | 1.07 (1.01–1.13) | 0.032 |
|  |  |  |  |  |  |  |  |  |  |  | RFS multi | 1.12 (0.97–1.47) | 0.156 |
| 38 | Kotha NV ^61^ | 2021 | USA | LSCC | Med -52 months | 1047 |  | T3-4, N0-N3 | Contal & O’Quigley cutpoint selection method  & Cox Wald cutpoint selection method | 4.17 | OS multi | 1.31 (1.12–1.54) | 0.001 |
|  |  |  |  |  |  |  |  |  |  |  | CSS multi | 1.46 (1.17–1.83) | <.001 |
| 39 | Kreinbrink PJ ^62^ | 2021 | United States | OPSCC | 2007–2018, Med-40 months | 201 | HPV +/- | I-IV, N0-N3 | Literature | 3 | OS multi | 1.49 (0.86, 2.59) | 0.16 |
|  |  |  |  |  |  |  |  |  |  |  | PFS multi | 1.66 (1.03, 2.69) | 0.039 |
| 40 | Kuo C ^63^ | 2019 | Taiwan | HPSCC | 09/2009–10/2015,  Med- 24.1 months (3.1–111.3) | 120 |  | I-IV |  | 4 | OS uni | 2.10 (1.23–3.58) | <0.01 |
|  |  |  |  |  |  |  |  |  |  |  | OS multi | 1.99 (1.21–3.28) | 0.01 |
|  |  |  |  |  |  |  |  |  |  |  | PFS uni | 1.80 (1.06–3.04) | 0.01 |
|  |  |  |  |  |  |  |  |  |  |  | PFS multi | 1.71 (1.01–2.90) | 0.046 |
| 41 | Kuwahara T ^64^ | 2018 | Japan | HPSCC | 12/2005–12/2015,  Med-38 months (2–101) | 111 |  | T1-4, III-IV, N0-N3 | ROC curve | 3.59 | OS uni | 2.39 (1.17–4.86) | 0.018 |
|  |  |  |  |  |  |  |  |  |  |  | OS multi | 2.30 (1.12–4.71) | 0.024 |
|  |  |  |  |  |  |  |  |  |  |  | PFS uni | 2.37 (1.30–4.26) | 0.005 |
|  |  |  |  |  |  |  |  |  |  |  | PFS multi | 2.12 (1.15–3.86) | 0.017 |
| 42 | Lee S ^65^ | 2020 | Korea | OSCC | 11/2005–08/2018,  Mean -41 months **(**3–144) | 291 |  | T1-4, I-IV, N0-N3 | ROC curve | OS-2.23, DFS-2.16 | OS uni | 2.01 (1.15–3.53) | 0.015 |
|  |  |  |  |  |  |  |  |  |  |  | OS multi | 1.78 (1.01–3.14) | 0.045 |
|  |  |  |  |  |  |  |  |  |  |  | DFS uni | 2.10 (1.39–3.15) | <0.001 |
|  |  |  |  |  |  |  |  |  |  |  | DFS multi | 1.82 (1.12–2.94) | 0.015 |
| 43 | Li L ^66^ | 2022 | China | OTSCC | 08/2009–04/2022 | 224 |  | T1-3, I-IV, N0-N3 | ROC curve | 1.830 | OS uni | 2.504 (1.701–3.688) | <0.001 |
|  |  |  |  |  |  |  |  |  |  |  | OS multi | 2.244 (1.314–3.830) | 0.003 |
| 44 | Li XH ^67^ | 2017 | China | NPC | 12/2006–07/2016 | 249 (validation set) |  | T1-4, I-IV, N0-N3 | ROC curve | 2.5 | DSS uni | 3.438 (2.008–5.888) | <0.001 |
|  |  |  |  |  |  |  |  |  |  |  | DSS multi | 1.939 (1.004–3.761) | 0.049 |
| 45 | Li Z ^68^ | 2021 | China | LSCC | 01/2008–12/2018,  Mean ± SD - 54.2 ± 31.9 (3–101) | 147 |  | T1-4, I-III, N0-N3 | ROC curve | 1.88 | OS uni | 4.487 (2.400–8.389) | <.001 |
|  |  |  |  |  |  |  |  |  |  |  | OS multi | 4.359 (2.310–8.227) | <.001 |
|  |  |  |  |  |  |  |  |  |  |  | PFS uni | 3.420 (1.991–5.873) | <.001 |
|  |  |  |  |  |  |  |  |  |  |  | PFS multi | 3.291 (1.899–5.702) | <.001 |
| 46 | Liao JL ^69^ | 2018 | Taiwan | NPC | 01/2007–12/2013,  Med -4.4 years | 180 |  | T1-4, I-IV, N0-N3 | Literature | 3.6 | OS uni | 1.89 (1.08–3.29) | 0.03 |
|  |  |  |  |  |  |  |  |  |  |  | OS multi | 2.76 (1.34–5.68) | 0.01 |
|  |  |  |  |  |  |  |  |  |  |  | PFS uni | 1.38 (0.76–2.48) | 0.12 |
|  |  |  |  |  |  |  |  |  |  |  | PFS multi | 1.76 (1.01–3.07) | 0.05 |
| 47 | Liew KY ^70^ | 2018 | Malaysia | NPC | 01/01/2005 - 31/12//2009, Mean ± SD - 64.85 ± 32.28 months | 98 |  | I-IV | ROC curve | 2.995 | DFS multi | 3.953 (1.896–8.240) | 0.00024 |
| 48 | Liu J ^71^ | 2020 | China | NPC | 01/2012–07/2019,  Med - 82 months | 207 |  | T1-4, I-IV, N0-N3 | ROC value | 2.49 | OS uni | 1.908 (2.135–2.860) | 0.029 |
|  |  |  |  |  |  |  |  |  |  |  | OS multi | 0.972 (1.276–1.982) | 0.081 |
| 49 | Lo WC ^72^ | 2017 | Taiwan | HPSCC | 2001-2008, Mean ± SD - 50.0 ± 34.7 months (4–140) | 105 |  | T3-4, N0-N3 | Median value | 3.22 | OS uni | 2.29 (1.37–3.84) | 0.001 |
|  |  |  |  |  |  |  |  |  |  |  | OS multi | 2.53 (1.48–4.30) | 0.001 |
|  |  |  |  |  |  |  |  |  |  |  | DSS uni | 2.22 (1.27–3.88) | 0.004 |
|  |  |  |  |  |  |  |  |  |  |  | DSS multi | 2.45 (1.38–4.34) | 0.002 |
|  |  |  |  |  |  |  |  |  |  |  | DFS uni | 2.19 (1.26–3.81) | 0.004 |
|  |  |  |  |  |  |  |  |  |  |  | DFS multi | 2.18 (1.24–3.83) | 0.007 |
| 50 | Lu A ^73^ | 2017 | China | NPC | Med-68 months (5–77) | 140 |  | T1-4, I-IV, N0-N3 | ROC value | 2.28 | OS multi | 2.383 (1.041–5.457) | 0.040 |
|  |  |  |  |  |  |  |  |  |  |  | PFS multi | 2.615 (1.206–5.672) | 0.015 |
| 51 | Lu ZY ^74^ | 2020 | China | OTSCC | 03/2012–11/2019 | 120 |  | T1-4, I-IV, N0-N3 | ROC value | 2.8 | OS uni | 3.264 (1.565–6.807) | 0.002 |
|  |  |  |  |  |  |  |  |  |  |  | OS multi |  | 0.165 |
|  |  |  |  |  |  |  |  |  |  |  | DFS uni | 2.417 (1.195–4.891) | 0.014 |
|  |  |  |  |  |  |  |  |  |  |  | DFS multi |  | 0.595 |
| 52 | Mittal S ^75^ | 2020 | India | HPSSC | Med-19 months (1.13–41.53) | 79 |  | T1-4, II-IV, N0-N3 | Literature | 3 | OS uni | 1.874 (1.0273.417) | 0.037 |
|  |  |  |  |  |  |  |  |  |  |  | OS multi | 1.088 (0.5342.214) | 0.817 |
|  |  |  |  |  |  |  |  |  |  |  | DFS uni | 1.895 (1.0723.349) | 0.025 |
|  |  |  |  |  |  |  |  |  |  |  | DFS multi | 1.194 (0.6142.321) | 0.602 |
| 53 | Moon H ^76^ | 2016 | Korea | HNSCC | Med-39.5 months (4.7–62.6) | 153 |  | T1-4, I-IV, N0-N3 |  | 2.6 | OS multi | 3.22 (1.41–7.09) | 0.005 |
|  |  |  |  |  |  |  |  |  |  |  | PFS multi | 2.20 (1.13–4.29) | 0.020 |
|  |  |  |  |  |  |  |  |  |  |  | CSS multi | 4.13 (1.57–9.19) | 0.003 |
| 54 | Muhaxheri G ^77^ | 2018 | Croatia | HNSCC | Mean -102.1 months | 182 |  | I-IV |  | 2.27 | OS multi | 1.375 (1.128–1.676) | 0.002 |
| 55 | Ng SP ^78^ | 2021 | United States | OPSSC | 2002–2013,  Med –59 months (6–153) | 848 | HPV +/- | T1-4, I-IV, N0-N3 |  | 3 | OS uni | 1.81 (1.35–2.42) | <0.0001 |
|  |  |  |  |  |  |  |  |  |  |  | OS multi | 1.64, (1.22–2.19) | 0.001 |
| 56 | Oka T ^79^ | 2023 | Japan | OPSCC | 01/2010–12/2018,  Med-5.3 years  (1.1–10.5) | 124 | HPV +/- | T1-4, I-IV, N0-N3 | ROC curve | 1.65 | OS uni | 3.78 (0.90–15.80) | 0.068 |
|  |  |  |  |  |  |  |  |  |  |  | OS multi | 3.62 (0.81–16.21) | 0.092 |
|  |  |  |  |  |  |  |  |  |  |  | DFS uni | 1.38 (0.62–3.05) | 0.433 |
|  |  |  |  |  |  |  |  |  |  |  | DSS uni | 4.28 (0.58–32.0) | 0.156 |
| 57 | Ong HS ^80^ | 2017 | China | OTSCC | Med-52 months (7–72) | 133 |  | T1-4 |  | 1,84 [(1,76x27,1) +72,9x1,88)] | OS uni | 2.02 (1.467–2.782) | <.001 |
|  |  |  |  |  |  |  |  |  |  |  | OS multi | 1.585 (1.016–2.754) | 0.102 |
|  |  |  |  |  |  |  |  |  |  |  | DFS uni | 2.036 (1.643–2.523) | <.001 |
|  |  |  |  |  |  |  |  |  |  |  | DFS multi | 1.535 (0.955–2.468) | 0.077 |
| 58 | Ono T ^81^ | 2018 | Japan | HPSCC | 2000–2014 | 96 |  | T1-4, III-IV, N0-N3 | Median value | 2.31 | OS uni | 1.03 (0.60–1.79) | 0.912 |
| 59 | Ooyama T ^82^ | 2025 | Japan | OSCC | 01/2017–03/2023 | 42 |  |  | ROC | 6.4 | OS (multi) | 0.83 (0.29–2.38) | 0.72 |
|  |  |  |  |  |  |  |  |  |  |  | PFS (uni) | 2.13 (1.08–4.35) | 0.022 |
|  |  |  |  |  |  |  |  |  |  |  | PFS (multi) | 0.87 (0.31–2.44) | 0.79 |
| 60 | Park MJ ^83^ | 2018 | Korea | HNSCC | 09/2010–08/2015,  Med-41 months (20–65) | 310 |  | T1-4, III-IV, N0-N3 | ROC curve | 2.5 | OS uni | 1.84 (1.10–3.08) | 0.020 |
|  |  |  |  |  |  |  |  |  |  |  | DFS uni | 1.54 (0.90–2.64) | 0.117 |
| 61 | Perisanidis C ^84^ | 2013 | Austria | OPSCC | 2001–2009,  Med - 3.7 years (0.3–9.5) | 97 |  | III-IV | ROC curve | 1.9 | DSS multi | 10.37 (1.28–84.08) | 0.029 |
| 62 | Rey M ^85^ | 2025 | France | HNSCC | 1/1/2017- 30/6/2021 | 112 | p16+ - 18 (16.1%) | T1-4 Tx, I-IV, N0-N3 | Literature | 3.5 | OS (multi) | 0.50 (0.23–1.10) | 0.085 |
| 63 | Ruiz-Ranz M ^86^ | 2022 | Spain | OSCC | Med- 54 months | 348 |  | T1-4, I-IV, N0-N3 | 90th percentile | 4.08 | OS uni | 1.847 (1.179–2.895) | 0.007 |
|  |  |  |  |  |  |  |  |  |  |  | OS multi | 1.626 (1.004–2.633) | 0.04 |
|  |  |  |  |  |  |  |  |  |  |  | DSS uni | 1.644 (0.956–2.829) | 0.07 |
| 64 | Sano Y ^87^ | 2018 | Japan | OSCC | 2007–2015, Med- 41.6 months (3.0–107.6) | 94 |  | T1-4, III-IV, N0-N2 | ROC curve | 2.36 | OS uni | 2.17 (1.03–4.57) | 0.04 |
|  |  |  |  |  |  |  |  |  |  |  | CSS uni | 2.63 (1.13–6.10) | 0.02 |
| 65 | Sheng X ^88^ | 2019 | China | LSCC | 01/2008–06/2015 | 110 |  | T1-4, I-IV, N0-N3 | ROC curve | 2.22 | OS multi | 2.405 (0.968–5.974) | 0.059 |
| 66 | Shi J ^89^ | 2025 | China | HNSCC | 01/2020 - 30/12/2023 | 545 |  | T1-4, N0-N3 | Maximally selected rank statistics | 3.21 | OS (uni) | 3.964 (2.667–5.892) | <0.001 |
|  |  |  |  |  |  |  |  |  |  |  | OS (multi) | 1.612 (0.793–3.275) | 0.187 |
|  |  |  |  |  |  |  |  |  |  |  | DFS (uni) | 2.914 (2.061–4.121) | <0.001 |
|  |  |  |  |  |  |  |  |  |  |  | DFS (multi) | 1.388 (0.749–2.570) | 0.298 |
| 67 | So YK ^90^ | 2018 | Korea | OPSCC | 11/2004–12/2016 | 104 | HPV+ | T1-4, I-IV, N1-N2 | ROC curve | 2.42 | OS multi | 3.32 (0.58–19.15) | 0.179 |
|  |  |  |  |  |  |  |  |  |  |  | DFS multi | 4.16 (1.24–13.95) | 0.021 |
| 68 | Song S ^91^ | 2022 | China | NPC | 01/2013–12/2016 | 111 |  | I-IV, N0-N+ | ROC curve | 2.02 | OS multi | 8.480 (1.693–42.461) | 0.009 |
|  |  |  |  |  |  |  |  |  |  |  | PFS multi | 1.298 (0.550–3.063) | 0.552 |
| 69 | Song Y ***^92^ | 2024 | China | OSCC | 04/2014–12/2021 | 58 |  | T1-4, I-IV, N0-NX | ROC& X-tile software | 2.12 | OS (uni) | 2.93 (1.02–8.41) | 0.046 |
|  |  |  |  |  |  |  |  |  |  |  | PFS (uni) | 2.01 (0.88–4.61) | 0.099 |
| 70 | Sun W ^93^ | 2016 | China | NPC | 01/2008–12/2011, Median-50 months (5–84) | 251 |  | T1-4, I-IV, N0-N3 | ROC curve | OS- 2.6, PFS- 2.7 | OS uni | 2.41 (1.20–4.83) | 0.013 |
|  |  |  |  |  |  |  |  |  |  |  | OS multi | 1.87 (0.89–3.95) | 0.099 |
|  |  |  |  |  |  |  |  |  |  |  | PFS uni | 2.78 (1.81–4.27) | 0.001 |
|  |  |  |  |  |  |  |  |  |  |  | PFS multi | 2.01 (1.23–3.29) | 0.005 |
| 71 | Sun W ^94^ | 2020 | China | OSCC | 04/2014–01/2020, Med-38 months (4–84) | 111 |  | T1-4, N0-N+ | ROC curve | 3.1 | OS uni | 2.39 (1.24–4.61) | 0.009 |
|  |  |  |  |  |  |  |  |  |  |  | OS multi | 2.08 (1.03–4.19) | 0.040 |
| 72 | Sunkara PR ^95^ | 2025 | USA | HNSCC | 22/01/2007 - 29/03/2013 | 681 | HPV+ OP - 57 (8.4), HPV−/unknown OP - 74 (10.8%) | T1-4, N0-N3 NX | Literature | 3 | OS (uni) | 1.09 (0.81–1.47) |  |
|  |  |  |  |  |  |  |  |  |  |  | DFS (uni) | 1.08 (0.84–1.39) |  |
| 73 | Tazeen S ^96^ | 2020 | India | OSCC | 2016–2019 | 130 |  | T1-4, I-IV, N0-N3 | ROC curve | 3.1 | OS multi | 1.171 (0.449–3.053) | 0.747 |
| 74 | Tham T ^97^ | 2019 | United States | HNSCC | 2008–2016 | 123 | HPV +/- | T1-4, N0-N3 | ROC curve | 2.87 | OS uni | 3.46 (1.12–10.68) | 0.031 |
| 75 | Tomasoni M ^98^ | 2023 | Italy | HNSCC | 03/2004–06/2018 | 542 |  | T1-4, II-IV, N0-N3 | x-tile programme (cut-off values with lowest P value) | OS- 4.2, RFS- 3.3 | OS uni |  | 0.024 |
|  |  |  |  |  |  |  |  |  |  |  | OS multi | 1.61 (1.17–2.21) | 0.003 |
|  |  |  |  |  |  |  |  |  |  |  | RFS uni |  | 0.381 |
|  |  |  |  |  |  |  |  |  |  |  | RFS multi | 1.47 (1.08–2.00) | 0.013 |
| 76 | Trevisani LF ^99^ | 2023 | Brazil | OSCC | 10/2009 - 12/2018, Mean ± SD 33.1 ± 27 months (0–133) | 600 |  | T1-4, N0-N2 | ROC curve | 3.38 | OS uni | 1.568 (1.231–1.998) | <0.001 |
|  |  |  |  |  |  |  |  |  |  |  | OS multi | 1.315 (0.983–1.761) | 0.065 |
| 77 | Tsai YT ^100^ | 2023 | Taiwan | OSCC | 01/2008-31/12/2019,  Med 40.1 months (3.5–122.4) | 288 |  | T1-4, I-IV, N0-N3 | ROC curve | 4.51 | OS uni | 4.465 (2.762–7.219) | <0.001 |
|  |  |  |  |  |  |  |  |  |  |  | OS multi | 2.710 (1.783–4.118) | <0.001 |
| 78 | Tsai YT ^101^ | 2022 | Taiwan | OSCC | 01/2007–12/2019 | 303 |  | T1-4, I-IV, N0-N3 | ROC curve | 4.51 | OS uni | 4.212 (2.621–6.770) | <0.001 |
|  |  |  |  |  |  |  |  |  |  |  | OS multi | 2.339 (1.365–4.007) | 0.002 |
|  |  |  |  |  |  |  |  |  |  |  | DFS uni | 2.500 (1.653–3.781) | <0.001 |
|  |  |  |  |  |  |  |  |  |  |  | DFS multi | 1.787 (1.131–2.825) | 0.013 |
| 79 | Tu XP ^102^ | 2015 | China | LSCC | Med-51 months (5–102) | 141 |  | T1-4, I-IV, N0-N+ | ROC | 2.17 | OS multi | 2.177 (1.208–3.924) | 0.010 |
|  |  |  |  |  |  |  |  |  |  |  | DFS multi | 1.869 (1.078–3.243) | 0.026 |
| 80 | Valdes V ^103^ | 2020 | Canada | HNSCC | Med -2.7 years (1.9–3.4) | 118 | HPV +/- |  | Significant association at 5 by univariate analysis | 5 | OS multi | 2.995 (1.174–7.641) | 0.055 |
| 81 | Wakisaka R ^104^ | 2024 | Japan | HNSCC | 04/2014–12/2018 | 83 |  | T1-4, III-IV, N0-N3 | ROC curve | OS - 3.81, PFS - 3.355 | OS (uni) | 2.53 (1.15–5.56) | 0.007 |
|  |  |  |  |  |  |  |  |  |  |  | OS (multi) | 0.71 (0.41–1.15) | 0.19 |
|  |  |  |  |  |  |  |  |  |  |  | PFS (uni) | 1.68 (0.88–3.19) | 0.083 |
| 82 | Wang CC ^105^ | 2024 | Taiwan | HNSCC | 07/2008–05/2021, Med- 6.1 years (0.3–12) | 614 |  | I-IV | ROC curve | 2.5 | OS multi | 1.25 (0.87–1.80) | 0.219 |
| 83 | Wang S ^106^ | 2022 | China | HNSCC | 01/2017–02/2021, Med- 31.7 months (11.0–46.4) | 155 | HPV +/- | T1-4, I-IV, N0-N3 | ROC curve | 2.46 | OS uni |  | <0.001 |
|  |  |  |  |  |  |  |  |  |  |  | OS multi | 3.690 (1.955–6.963) | <0.001 |
|  |  |  |  |  |  |  |  |  |  |  | PFS uni |  | <0.001 |
|  |  |  |  |  |  |  |  |  |  |  | PFS multi | 3.163 (1.810–5.528) | <0.001 |
| 84 | Wang WY*** ^107^ | 2024 | China | HNSCC | 04/2008–12/2017 | 181 |  | T1-4, I-IV, N0-N3 | ROC curve | 3.21 | OS (uni) | 1.11 (1.06–1.15) | <0.001 |
|  |  |  |  |  |  |  |  |  |  |  | RFS (uni) | 1.12 (1.07–1.17) | 0.001 |
| 85 | Watabe Y ^108^ | 2021 | Japan | OSCC | 01/2004–12/2012, Med- 68 months (50–96) | 110 |  | I-IV | ROC curve | 1.788 | OS uni | 5.42 (1.26–23.36) | 0.023 |
|  |  |  |  |  |  |  |  |  |  |  | OS multi | 1.02 (0.17–6.08) | 0.981 |
|  |  |  |  |  |  |  |  |  |  |  | DFS uni | 2.95 (1.23–7.12) | 0.016 |
|  |  |  |  |  |  |  |  |  |  |  | DFS multi | 1.79 (0.66–4.84) | 0.250 |
| 86 | Wu CN ^109^ | 2017 | Taiwan | OTSCC | 2004–2015, 67.1 months  (2–137) | 262 |  | T1-2, N0-N+ | ROC curve | 2.95 | OS multi | 2.292 (1.326–3.962) | 0.003 |
|  |  |  |  |  |  |  |  |  |  |  | DSS multi | 2.106 (1.007–4.405) | 0.048 |
|  |  |  |  |  |  |  |  |  |  |  | DFS multi | 1.914 (1.02–3.595) | 0.043 |
| 87 | Xun Y ^110^ | 2020 | China | LSCC | 04/2008–03/2019 | 151 |  | T1-4, I-IV, N0-N2 | ROC curve | 2.2 | OS uni | 0.22 (0.12–0.41) | <.001 |
|  |  |  |  |  |  |  |  |  |  |  | OS multi | 3.02 (1.28–7.10) | 0.011 |
|  |  |  |  |  |  |  |  |  |  |  | PFS uni | 0.17 (0.85–0.33) | <.001 |
|  |  |  |  |  |  |  |  |  |  |  | PFS multi | 3.56 (1.47–8.62) | 0.016 |
| 88 | Yamahara K ^111^ | 2021 | Japan | HNSCC | Med-53 months (2–125) | 164 |  | T1-4, I-IV, N0-N3 | ROC curve | 4.2 | OS uni | 0.406 (0.177–0.930) | 0.03 |
|  |  |  |  |  |  |  |  |  |  |  | OS multi | 0.87 (0.23–3.28) | 0.84 |
| 89 | Yang J ^112^ | 2018 | China | HPSCC | 12/2009–12/2017,  Med-30.95 months (1–82) | 197 |  | T1-4, I-IV, N0-N3 | X-tile programme (cutoff points with minimum p values) | 2.69 | OS uni | 1.49 (1.02–2.18) | 0.040 |
|  |  |  |  |  |  |  |  |  |  |  | DFS uni | 1.60 (1.11–2.31) | 0.012 |
|  |  |  |  |  |  |  |  |  |  |  | CSS uni | 1.54 (1.04–2.29) | 0.031 |
|  |  |  |  |  |  |  |  |  |  |  | OS multi | 0.95 (0.63–1.43) | 0.796 |
| 90 | Yanni A ^113^ | 2022 | Belgium | HNSCC | 2000–2017, Med - 29.5 months (12.5–57.5) | 95 |  | T1-4, N0-N3 | X-tile programme (cutoff points with minimum p values) | 2**.77** | OS uni | 2.39 (1.24–4.61) | 0.009 |
|  |  |  |  |  |  |  |  |  |  |  | OS multi | 1.3 (1.04–1.62) | 0.024 |
| 91 | Yao JJ ^114^ | 2019 | China | NPC | Med- 54.3 months (1.3–85.6) | 1550 |  | T1-4, II-IV, N0-N3 | ROC curve | 2.5 | OS multi | 1.72 (1.31–2.24) | < 0.001 |
|  |  |  |  |  |  |  |  |  |  |  | DMFS multi | 1.45 (1.10–1.92) | 0.009 |
|  |  |  |  |  |  |  |  |  |  |  | PFS multi | 1.29 (1.04–1.59) | 0.021 |
| 92 | Ye J ^115^ | 2020 | China | HNSCC | Med ± SD - 51.7 ± 0.34 months | 197 |  | T1-4, N0-N2 | ROC curve | 2.77 | OS uni | 1.1 (1.0–1.1) | 0.001 |
| 93 | Ye L ^116^ | 2018 | China | NPC | Med-67.5 months (4.8–85.5) | 427 |  | T1-4, I-IV, N0-N3 | Median value | 2.32 | OS multi (unadjusted) | 1.872 (1.118–3.137) | 0.017 |
|  |  |  |  |  |  |  |  |  |  |  | OS multi (adjusted) | 1.699 (1.005–2.873) | 0.048 |
|  |  |  |  |  |  |  |  |  |  |  | PFS multi (unadjusted) | 1.747 (1.181–2.585) | 0.005 |
|  |  |  |  |  |  |  |  |  |  |  | PFS multi (Adjusted) | 1.710 (1.150–2.543) | 0.008 |
| 94 | Yun JM ^117^ | 2023 | Korea | OPSCC | 03/1994–03/2019, Med -1102 days (444.0–1964.5) | 461 |  | T1-4, I-IV, N0-N3 | ROC curve | 2.37 | OS uni | 2.77 (1.62–4.73) | <0.001 |
|  |  |  |  |  |  |  |  |  |  |  | OS multi | 2.19 (1.17–4.08) | 0.014 |
|  |  |  |  |  |  |  |  |  |  |  | RFS uni | 1.75 (1.21–2.51) | 0.003 |
|  |  |  |  |  |  |  |  |  |  |  | RFS multi | 1.65 (1.08–2.52) | 0.022 |
| 95 | Zeng X ^118^ | 2020 | China | NPC | 01/2014–02/2019, Med 33.5 months (2.1–151.2) | 507 |  | T1-4, N0-N3 | ROC curve | 3.441 | OS uni | 4.005 (1.633–9.820) | 0.002 |
|  |  |  |  |  |  |  |  |  |  |  | OS multi | 2.354 (0.507–10.93) | 0.275 |
| 96 | Zeng YC ^119^ | 2016 | China | LSCC | 01/2007–09/2013, Med-45 months (3–66) | 115 |  | T3-4, N0-N3 | Median value | 3 | OS uni | 1.55 (1.07–2.25) | 0.020 |
|  |  |  |  |  |  |  |  |  |  |  | OS multi | 1.51 (1.04–2.20) | 0.029 |
|  |  |  |  |  |  |  |  |  |  |  | PFS uni | 1.52 (1.05–2.21) | 0.026 |
|  |  |  |  |  |  |  |  |  |  |  | PFS multi | 1.79 (1.21–2.64) | 0.003 |
| 97 | Zhong B ^120^ | 2019 | China | SNSCC | 05/2008 - 10/2017, Med ± SD - 39.21 ± 15.36 months | 147 |  | T1-4, N0-N3 | Median value | 4.25 | OS uni | 1.888 (1.336–3.342) | <0.001 |
|  |  |  |  |  |  |  |  |  |  |  | OS multi | 1.579 (1.217–3.092) | 0.002 |
|  |  |  |  |  |  |  |  |  |  |  | DFS uni | 1.763 (1.156–3.149) | <0.001 |
|  |  |  |  |  |  |  |  |  |  |  | DFS multi | 1.688 (1.162–3.363) | <0.001 |
|  |  |  |  |  |  |  |  |  |  |  | DSS uni | 1.359 (1.033–3.314) | <0.001 |
|  |  |  |  |  |  |  |  |  |  |  | DSS multi | 1.509 (1.153–3.104) | 0.001 |
| 98 | Zhou J ^121^ | 2023 | China | HNSCC | 01/2018–12/2021, Med 24 months (1–47) | 304 |  | T1-4, I-IV, N0-N3 | ROC curve | 1.94 | OS uni | 1.176 (1.120–1.235) | 0.001 |
|  |  |  |  |  |  |  |  |  |  |  | OS multi | 1.104 (1.016–1.20) | 0.019 |
| 99 | Zhou S ^122^ | 2020 | China | HNSCC | 06/2015–06/2019, Med-27.2 months (2–48) | 367 |  | T1-4, I-IV, N0-N2 | ROC curve | 2.81 | OS uni | 2.151 (1.363–3.394) | 0.001 |
|  |  |  |  |  |  |  |  |  |  |  | OS multi | 0.692 (0.329–1.456) | 0.332 |
|  |  |  |  |  |  |  |  |  |  |  | DFS uni | 3.371 (2.490–4.563) | 0.001 |
|  |  |  |  |  |  |  |  |  |  |  | DFS multi | 1.731 (1.083–2.767) | 0.022 |
| 100 | Zubair F ^123^ | 2022 | United Kingdom | OSCC | 15/02/2006 - 16/12/2019 | 825 |  | N0-N3 | ROC curve | 5 | OS uni | 2.35 (1.78 to 3.09) | <0.001 |
|  |  |  |  |  |  |  |  |  |  |  | DSS uni | 2.37 (1.63 to 3.43) | <0.001 |

The reference numbers in the table correspond to those listed in the main article's reference list. (* HPV negative cohort; ** HPV positive cohort; *** Training cohort)

**TABLE S4. Included studies in the systematic review for evaluating the prognostic significance of tumor associated neutrophils (TANs) in HNSCC patients**

| **No.** | **First author and reference number** | **Year** | **Country** | **Tumor site** | **Study period / Median (IQR)** | **No. cases** | **HPV /EBV** | **Size** | **TANs cut-off method** | **TANs cut-off values or relevant data** | **Survival endpoint** | **HR (95 % CI)** | **P** |
| --- | --- | --- | --- | --- | --- | --- | --- | --- | --- | --- | --- | --- | --- |
| 1 | Brunkhorst H ^124^ | 2024 | Germany | HNSCC | 1998–2015, Med-4.3 years | 397 | HPV +/- | T1-4, I-IV, N0-N3 | Xtile software (lowest p values) | Stromal compartment neutrophils- (mean 372 neutrophils/mm^2^, SD ± 812) epithelial cancer compartment neutrophils- (mean 1040 neutrophils/mm^2^ ± 1477) | **HNSCC subgroup 1,4,5** | | |
|  |  |  |  |  |  |  |  |  |  |  | OS uni (Neutrophil epithelial) | 1.528 (0.855–2.731) | 0.002 |
|  |  |  |  |  |  |  |  |  |  |  | OS multi (Neutrophil epithelial) | 2.011 (1.29–3.135) | 0.002 |
|  |  |  |  |  |  |  |  |  |  |  | OS uni (Neutrophil stromal) | 1.415 (0.783–0.558) | 0.25 |
|  |  |  |  |  |  |  |  |  |  |  | OS multi (Neutrophil stromal) | 1.713 (0.978–3.001) | 0.060 |
|  |  |  |  |  |  |  |  |  |  |  | **HNSCC subgroup 2,3** | | |
|  |  |  |  |  |  |  |  |  |  |  | OS uni (Neutrophil epithelial) | 0.532 (0.282–1.003) | 0.051 |
|  |  |  |  |  |  |  |  |  |  |  | OS multi (Neutrophil epithelial) | 0.50 (0.27–0.926) | 0.027 |
|  |  |  |  |  |  |  |  |  |  |  | OS uni (Neutrophil stromal) | 0.537 (0.27–1.069) | 0.077 |
|  |  |  |  |  |  |  |  |  |  |  | OS multi (Neutrophil stromal) | 0.530 (0.281–1) | 0.050 |
| 2 | Li C ^125^ | 2019 | China | OSCC | 01/2007–12/2011 | 81 | HPV +/- | I-IV, N0-N+ |  | Median – CD15^+^ neutrophils-41.0 | Cancer-related survival Uni | 0.199 (0.085–0.465) | <0.001 |
|  |  |  |  |  |  |  |  |  |  |  | Cancer-related survival Multi | 3.078 (1.196–7.922) | 0.020 |
| 3 | Caldeira PC ^126^ | 2015 | Brazil | OSCC | Mean -29  (1–92 months) | 28 |  | T1-4 |  | CD66b infiltration and CD66b/CD3 ratio |  |  |  |
| 4 | Zhu X ^127^ | 2024 | China | LSCC | 06/2014–09/2017 (36 months) (cohort 1) | 61 |  | T1-T4, I-IV, N0, N1-N2 | Median | Intratumoral neutrophil count (CD66b^+^CD45^+^ cells) per spot (>50 median level) | OS (uni) | 4.637(1.346-15.976) | 0.015 |
|  |  |  |  |  |  |  |  |  |  |  | OS (multi) | 2.949(0.790-11.013) | 0.108 |
|  |  |  |  |  | 09/2021–07/2023 (Cohort 2) | 50 |  | T1-T4, I-IV, N0, N1-N2 | Median percentage of CXCR4^+^ TANs | <20%, >20% (CXCR4^+^ neutrophil proportion in total CD66b^+^ neutrophils) | OS (multi) | 4.149(1.076-15.995) | 0.039 |
| 5 | Zhu X ^128^ | 2024 | China | HNSCC | 06/2014 - 09/2017 | 80 |  | T1-T4, I-III, N0, N1-N2 | X-tile software - Median (range)  CD66b^+^CD206^+^iNOS^−^ neutrophils and a few CD66b^+^CD206^+^iNOS^+^ neutrophils 🡪 HNSCC infiltrating N2 neutrophils.  CD66b^+^CD206^−^iNOS^+^ neutrophils and a few CD66b^+^CD206^−^iNOS^−^ neutrophils 🡪 HNSCC-infiltrating N1 neutrophils | Tumor Nest: - N1- 3 (0-18), N2- 3 (0-34), N2/N1 -0.59 (0-8); | OS (multi)- N1 | 0.355 (0.132–0.950) | 0.039 |
|  |  |  |  |  |  |  |  |  |  |  | OS (multi)- N2 | 3.069 (1.144–8.233) | 0.026 |
|  |  |  |  |  |  |  |  |  |  |  | OS (multi)- N2/N1 | 4.458(1.384–14.366) | 0.012 |
|  |  |  |  |  |  |  |  |  |  | Tumor Stroma: N1- 4 (1–22), N2-2 (0–14), N2/N1 - 0.30 (0-5); | OS (multi) - N1 | 0.874(0.351–2.173) | 0.772 |
|  |  |  |  |  |  |  |  |  |  |  | OS (multi) - N2 | 2.485(0.725–8.513) | 0.148 |
|  |  |  |  |  |  |  |  |  |  |  | OS (multi)- N2/N1 | 3.01(1.072–8.45) | 0.036 |
|  |  |  |  |  |  |  |  |  |  | Combined tumor area: - TAN1- 8 (1-31), TAN2- 4 (0-41), N2/N1 - 0.5 (0-4.2) | OS (multi) - N1 | 0.572(0.216–1.515) | 0.261 |
|  |  |  |  |  |  |  |  |  |  |  | OS (multi) - N2 | 1.61(0.54–4.794) | 0.393 |
|  |  |  |  |  |  |  |  |  |  |  | OS (multi)- N2/N1 | 3.373(1.269–8.965) | 0.015 |

The reference numbers in the table correspond to those listed in the main article's reference list.

**TABLE S5. Quality Analysis for NLR as a Prognostic Marker in HNSCC based on REMARK (C1-C6) and MASARATI Criteria (Q1-Q10)**

(Green circle- all necessary parameters are provided (sufficient): Yellow circle – one important parameter is missing (insufficient): Red circle – two or more parameters are missing (not eligible). Quality scores interpret study reliability: 90–100%- excellent reliability (low bias); 75–89%- good reliability (low bias); 50–74%- moderate reliability (moderate bias)- 25–49%: low reliability (high bias); 0–24%- very low reliability (high bias)

| No. | Study | C1 | C2 | C3 | C4 | C5 | C6 | C% | Risk of bias based on REMARK criteria | Q1 | Q2 | Q3 | Q4 | Q5 | Q6 | Q7 | Q8 | Q9 | Q10 | Q% | Risk of bias based on MASARIT criteria |
| --- | --- | --- | --- | --- | --- | --- | --- | --- | --- | --- | --- | --- | --- | --- | --- | --- | --- | --- | --- | --- | --- |
| 1 | Al-Rajhi, N. | 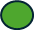 | 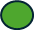 | 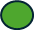 | 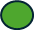 | 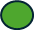 | 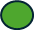 | 100% | Low | 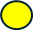 | 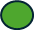 | 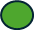 | N/A | 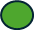 | 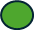 | 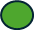 | 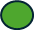 | 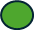 | 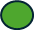 | 94% | Low |
| 2 | An, X | 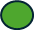 | 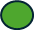 | 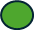 | 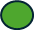 | 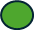 | 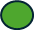 | 100% | Low | 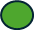 | 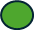 | 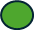 | N/A | 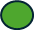 | 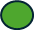 | 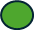 | 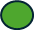 | 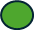 | 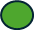 | 100% | Low |
| 3 | Ansarin, M. | 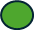 | 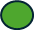 | 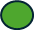 | 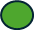 | 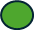 | 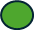 | 100% | Low | 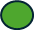 | 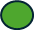 | 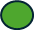 | N/A | 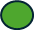 | 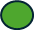 | 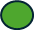 | 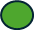 | 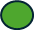 | 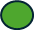 | 100% | Low |
| 4 | Bobdey. S | 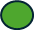 | 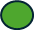 | 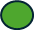 | 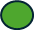 | 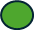 | 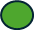 | 100% | Low | 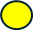 | 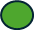 | 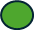 | N/A | 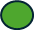 | 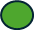 | 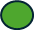 | 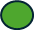 | 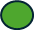 | 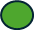 | 94% | Low |
| 5 | Brewczynski, A | 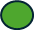 | 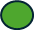 | 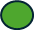 | 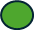 | 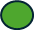 | 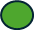 | 100% | Low | 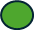 | 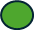 | 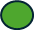 | N/A | 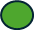 | 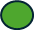 | 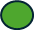 | 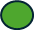 | 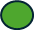 | 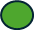 | 100% | Low |
| 6 | Brkic, F. F. | 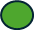 | 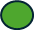 | 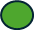 | 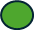 | 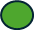 | 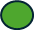 | 100% | Low | 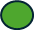 | 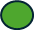 | 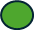 | N/A | 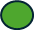 | 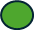 | 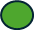 | 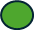 | 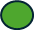 | 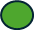 | 100% | Low |
| 7 | Cai, H. | 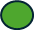 | 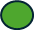 | 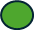 | 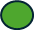 | 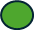 | 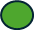 | 100% | Low | 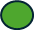 | 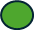 | 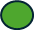 | N/A | 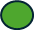 |  |  |  |  |  | 100% | Low |
| 8 | Charles, K.A. |  |  |  |  |  |  | 100% | Low |  |  |  | N/A |  |  |  |  |  |  | 100% | Low |
| 9 | Chen, L. |  |  |  |  |  |  | 100% | Low |  |  |  | N/A |  |  |  |  |  |  | 100% | Low |
| 10 | Chen, M.F. |  |  |  |  |  |  | 100% | Low |  |  |  | N/A |  |  |  |  |  |  | 100% | Low |
| 11 | Cho, U. |  |  |  |  |  |  | 100% | Low |  |  |  | N/A |  |  |  |  |  |  | 100% | Low |
| 12 | Cho, Y. |  |  |  |  |  |  | 100% | Low |  |  |  | N/A |  |  |  |  |  |  | 100% | Low |
| 13 | Chua, M.L. |  |  |  |  |  |  | 100% | Low |  |  |  | N/A |  |  |  |  |  |  | 100% | Low |
| 14 | De Almeida, J.R. |  |  |  |  |  |  | 100% | Low |  |  |  | N/A |  |  |  |  |  |  | 100% | Low |
| 15 | Du, J |  |  |  |  |  |  | 100% | Low |  |  |  | N/A |  |  |  |  |  |  | 94% | Low |
| 16 | Eskiizmir, G. |  |  |  |  |  |  | 100% | Low |  |  |  | N/A |  |  |  |  |  |  | 100% | Low |
| 17 | Fanetti, F. |  |  |  |  |  |  | 100% | Low |  |  |  | N/A |  |  |  |  |  |  | 100% | Low |
| 18 | Fang, Y.H. |  |  |  |  |  |  | 100% | Low |  |  |  | N/A |  |  |  |  |  |  | 100% | Low |
| 19 | Fu, Y |  |  |  |  |  |  | 100% | Low |  |  |  | N/A |  |  |  |  |  |  | 100% | Low |
| 20 | Go, J.Y. |  |  |  |  |  |  | 100% | Low |  |  |  | N/A |  |  |  |  |  |  | 100% | Low |
| 21 | Gorphe, P |  |  |  |  |  |  | 100% | Low |  |  |  | N/A |  |  |  |  |  |  | 100% | Low |
| 22 | Graupp, M. |  |  |  |  |  |  | 100% | Low |  |  |  | N/A |  |  |  |  |  |  | 94% | Low |
| 23 | Gundog, M |  |  |  |  |  |  | 100% | Low |  |  |  | N/A |  |  |  |  |  |  | 100% | Low |
| 24 | Haring, C.T. |  |  |  |  |  |  | 92% | Low |  |  |  | N/A |  |  |  |  |  |  | 94% | Low |
| 25 | Hasegawa, T |  |  |  |  |  |  | 100% | Low |  |  |  | N/A |  |  |  |  |  |  | 100% | Low |
| 26 | Homa-Mlak, I |  |  |  |  |  |  | 100% | Low |  |  |  | N/A |  |  |  |  |  |  | 100% | Low |
| 27 | Iglesias, L.J. |  |  |  |  |  |  | 100% | Low |  |  |  | N/A |  |  |  |  |  |  | 100% | Low |
| 28 | Ikeguchi, M |  |  |  |  |  |  | 100% | Low |  |  |  | N/A |  |  |  |  |  |  | 100% | Low |
| 29 | Iuchi, H. |  |  |  |  |  |  | 100% | Low |  |  |  | N/A |  |  |  |  |  |  | 100% | Low |
| 30 | Jin, Y. |  |  |  |  |  |  | 92% | Low |  |  |  | N/A |  |  |  |  |  |  | 94% | Low |
| 31 | Kano, S |  |  |  |  |  |  | 100% | Low |  |  |  | N/A |  |  |  |  |  |  | 100% | Low |
| 32 | Kao, H.K. |  |  |  |  |  |  | 100% | Low |  |  |  | N/A |  |  |  |  |  |  | 100% | Low |
| 33 | Kasahara, Y. |  |  |  |  |  |  | 92% | Low |  |  |  | N/A |  |  |  |  |  |  | 94% | Low |
| 34 | Kawano, T. |  |  |  |  |  |  | 100% | Low |  |  |  | N/A |  |  |  |  |  |  | 100% | Low |
| 35 | Kaźmierska, J |  |  |  |  |  |  | 100% | Low |  |  |  | N/A |  |  |  |  |  |  | 94% | Low |
| 36 | Khazravi, M. |  |  |  |  |  |  | 100% | Low |  |  |  | N/A |  |  |  |  |  |  | 100% | Low |
| 37 | Kim, D.Y. |  |  |  |  |  |  | 100% | Low |  |  |  | N/A |  |  |  |  |  |  | 100% | Low |
| 38 | Kotha, N.V. |  |  |  |  |  |  | 100% | Low |  |  |  | N/A |  |  |  |  |  |  | 100% | Low |
| 39 | Kreinbrink, P.J. |  |  |  |  |  |  | 100% | Low |  |  |  | N/A |  |  |  |  |  |  | 100% | Low |
| 40 | Kuo, C |  |  |  |  |  |  | 100% | Low |  |  |  | N/A |  |  |  |  |  |  | 100% | Low |
| 41 | Kuwahara, T |  |  |  |  |  |  | 100% | Low |  |  |  | N/A |  |  |  |  |  |  | 100% | Low |
| 42 | Lee, S |  |  |  |  |  |  | 100% | Low |  |  |  | N/A |  |  |  |  |  |  | 100% | Low |
| 43 | Li, L |  |  |  |  |  |  | 100% | Low |  |  |  | N/A |  |  |  |  |  |  | 100% | Low |
| 44 | Li, X.H |  |  |  |  |  |  | 100% | Low |  |  |  | N/A |  |  |  |  |  |  | 100% | Low |
| 45 | Li, Z |  |  |  |  |  |  | 100% | Low |  |  |  | N/A |  |  |  |  |  |  | 100% | Low |
| 46 | Liao, J.L. |  |  |  |  |  |  | 100% | Low |  |  |  | N/A |  |  |  |  |  |  | 100% | Low |
| 47 | Liew, K.Y. |  |  |  |  |  |  | 100% | Low |  |  |  | N/A |  |  |  |  |  |  | 100% | Low |
| 48 | Liu, J |  |  |  |  |  |  | 100% | Low |  |  |  | N/A |  |  |  |  |  |  | 100% | Low |
| 49 | Lo, W.C. |  |  |  |  |  |  | 100% | Low |  |  |  | N/A |  |  |  |  |  |  | 94% | Low |
| 50 | Lu, A |  |  |  |  |  |  | 100% | Low |  |  |  | N/A |  |  |  |  |  |  | 100% | Low |
| 51 | Lu, Z.Y. |  |  |  |  |  |  | 100% | Low |  |  |  | N/A |  |  |  |  |  |  | 100% | Low |
| 52 | Mittal, S. |  |  |  |  |  |  | 100% | Low |  |  |  | N/A |  |  |  |  |  |  | 100% | Low |
| 53 | Moon, H |  |  |  |  |  |  | 100% | Low |  |  |  | N/A |  |  |  |  |  |  | 100% | Low |
| 54 | Muhaxheri, G. |  |  |  |  |  |  | 100% | Low |  |  |  | N/A |  |  |  |  |  |  | 100% | Low |
| 55 | Ng, S.P. |  |  |  |  |  |  | 100% | Low |  |  |  | N/A |  |  |  |  |  |  | 100% | Low |
| 56 | Oka, T. |  |  |  |  |  |  | 92% | Low |  |  |  | N/A |  |  |  |  |  |  | 100% | Low |
| 57 | Ong, H.S. |  |  |  |  |  |  | 92% | Low |  |  |  | N/A |  |  |  |  |  |  | 100% | Low |
| 58 | Ono, T. |  |  |  |  |  |  | 100% | Low |  |  |  | N/A |  |  |  |  |  |  | 100% | Low |
| 59 | Ooyama, T. |  |  |  |  |  |  | 92% | Low |  |  |  | N/A |  |  |  |  |  |  | 94% | Low |
| 60 | Park, M.J. |  |  |  |  |  |  | 100% | Low |  |  |  | N/A |  |  |  |  |  |  | 100% | Low |
| 61 | Perisanidis, C. |  |  |  |  |  |  | 100% | Low |  |  |  | N/A |  |  |  |  |  |  | 100% | Low |
| 62 | Rey, M. |  |  |  |  |  |  | 83% | Low |  |  |  | N/A |  |  |  |  |  |  | 94% | Low |
| 63 | Ruiz-Ranz, M |  |  |  |  |  |  | 100% | Low |  |  |  | N/A |  |  |  |  |  |  | 100% | Low |
| 64 | Sano, Y |  |  |  |  |  |  | 100% | Low |  |  |  | N/A |  |  |  |  |  |  | 100% | Low |
| 65 | Sheng, X |  |  |  |  |  |  | 100% | Low |  |  |  | N/A |  |  |  |  |  |  | 100% | Low |
| 66 | Shi, J |  |  |  |  |  |  | 92% | Low |  |  |  | N/A |  |  |  |  |  |  | 100% | Low |
| 67 | So, Y.K. |  |  |  |  |  |  | 100% | Low |  |  |  | N/A |  |  |  |  |  |  | 100% | Low |
| 68 | Song, S. |  |  |  |  |  |  | 100% | Low |  |  |  | N/A |  |  |  |  |  |  | 100% | Low |
| 69 | Song, Y. |  |  |  |  |  |  | 100% | Low |  |  |  | N/A |  |  |  |  |  |  | 100% | Low |
| 70 | Sun, W. |  |  |  |  |  |  | 100% | Low |  |  |  | N/A |  |  |  |  |  |  | 100% | Low |
| 71 | Sun, W. |  |  |  |  |  |  | 100% | Low |  |  |  | N/A |  |  |  |  |  |  | 100% | Low |
| 72 | Sunkara, P.R. |  |  |  |  |  |  | 92% | Low |  |  |  | N/A |  |  |  |  |  |  | 100% | Low |
| 73 | Tazeen, S. |  |  |  |  |  |  | 100% | Low |  |  |  | N/A |  |  |  |  |  |  | 100% | Low |
| 74 | Tham, T |  |  |  |  |  |  | 100% | Low |  |  |  | N/A |  |  |  |  |  |  | 100% | Low |
| 75 | Tomasoni, M. |  |  |  |  |  |  | 100% | Low |  |  |  | N/A |  |  |  |  |  |  | 100% | Low |
| 76 | Trevisani, L.F. |  |  |  |  |  |  | 100% | Low |  |  |  | N/A |  |  |  |  |  |  | 100% | Low |
| 77 | Tsai, Y.T. |  |  |  |  |  |  | 100% | Low |  |  |  | N/A |  |  |  |  |  |  | 100% | Low |
| 78 | Tsai, Y.T. |  |  |  |  |  |  | 100% | Low |  |  |  | N/A |  |  |  |  |  |  | 100% | Low |
| 79 | Tu, X.P. |  |  |  |  |  |  | 100% | Low |  |  |  | N/A |  |  |  |  |  |  | 100% | Low |
| 80 | Valdes, V. |  |  |  |  |  |  | 100% | Low |  |  |  | N/A |  |  |  |  |  |  | 100% | Low |
| 81 | Wakisakw, R. |  |  |  |  |  |  | 100% | Low |  |  |  | N/A |  |  |  |  |  |  | 100% | Low |
| 82 | Wang, C.C. |  |  |  |  |  |  | 100% | Low |  |  |  | N/A |  |  |  |  |  |  | 94% | Low |
| 83 | Wang, S. |  |  |  |  |  |  | 100% | Low |  |  |  | N/A |  |  |  |  |  |  | 100% | Low |
| 84 | Wang, W.Y. |  |  |  |  |  |  | 83% | Low |  |  |  | N/A |  |  |  |  |  |  | 94% | Low |
| 85 | Watabe, Y |  |  |  |  |  |  | 100% | Low |  |  |  | N/A |  |  |  |  |  |  | 100% | Low |
| 86 | Wu, C.N. |  |  |  |  |  |  | 100% | Low |  |  |  | N/A |  |  |  |  |  |  | 100% | Low |
| 87 | Xun, Y. |  |  |  |  |  |  | 100% | Low |  |  |  | N/A |  |  |  |  |  |  | 100% | Low |
| 88 | Yamahara, K. |  |  |  |  |  |  | 100% | Low |  |  |  | N/A |  |  |  |  |  |  | 100% | Low |
| 89 | Yang, J. |  |  |  |  |  |  | 100% | Low |  |  |  | N/A |  |  |  |  |  |  | 100% | Low |
| 90 | Yanni, A |  |  |  |  |  |  | 100% | Low |  |  |  | N/A |  |  |  |  |  |  | 100% | Low |
| 91 | Yao, J.J. |  |  |  |  |  |  | 100% | Low |  |  |  | N/A |  |  |  |  |  |  | 100% | Low |
| 92 | Ye, J. |  |  |  |  |  |  | 100% | Low |  |  |  | N/A |  |  |  |  |  |  | 100% | Low |
| 93 | Ye, L. |  |  |  |  |  |  | 100% | Low |  |  |  | N/A |  |  |  |  |  |  | 100% | Low |
| 94 | Yun, J.M. |  |  |  |  |  |  | 100% | Low |  |  |  | N/A |  |  |  |  |  |  | 100% | Low |
| 95 | Zeng, X. |  |  |  |  |  |  | 100% | Low |  |  |  | N/A |  |  |  |  |  |  | 100% | Low |
| 96 | Zeng, Y.C. |  |  |  |  |  |  | 100% | Low |  |  |  | N/A |  |  |  |  |  |  | 100% | Low |
| 97 | Zhong, B. |  |  |  |  |  |  | 100% | Low |  |  |  | N/A |  |  |  |  |  |  | 100% | Low |
| 98 | Zhou, J. |  |  |  |  |  |  | 100% | Low |  |  |  | N/A |  |  |  |  |  |  | 100% | Low |
| 99 | Zhou, S. |  |  |  |  |  |  | 100% | Low |  |  |  | N/A |  |  |  |  |  |  | 100% | Low |
| 100 | Zubair, F. |  |  |  |  |  |  | 92% | Low |  |  |  | N/A |  |  |  |  |  |  | 88% | Low |

**TABLE S6. Results of Egger’s regression test for assessing publication bias in included studies**

| **Study category (survival endpoint and statistical data presentation)** | **Heterogeneity (I^2^)** | **Egger’s test, P-Value** | **Test–statistic (t)** | **Bias Estimate [SE -standard error of the estimated bias (intercept)]** | **No. of cohorts /studies** |
| --- | --- | --- | --- | --- | --- |
| OS - univariate | 89 % | < 0.0001 | 8.01 | 2.60 (SE = 0.3240) | 67 |
| OS - multivariate | 66 % | < 0.0001 | 5.81 | 1.56 (SE = 0.2693) | 76 |
| PFS - univariate | 76 % | 0.3082 | -1.06 | -1.81 (SE = 1.7032) | 14 |
| PFS - multivariate | 52 % | 0.0756 | 1.88 | 1.46 (SE = 0.7761) | 21 |
| DFS - univariate | 68 % | 0.9689 | -0.04 | -0.04 (SE = 0.8938) | 23 |
| DFS - multivariate | 51 % | 0.0052 | 3.31 | 2.69 (SE = 0.8132) | 16 |
| DSS - univariate | 50 % | - | - | - | 9^a^ |
| DSS - multivariate | 0% | - | - | - | 9^a^ |
| RFS - univariate | 53 % | - | - | - | 6^a^ |
| RFS - multivariate | 0 % | - | - | - | 5^a^ |

^a^ Unable to produce valid p-values or bias estimates in Egger’s test as the degree of freedom is less than 10.

**FIGURE S1.** Funnel plots of studies included in the meta-analysis evaluating the association between neutrophil-to-lymphocyte ratio (NLR) and survival outcomes, used to assess potential publication bias. Plots correspond to: A - Overall survival (OS), univariate data; B - OS, multivariate data; C - Progression-free survival (PFS), univariate data; D - PFS, multivariate data; E - Disease-free survival (DFS), univariate data; F - DFS, multivariate data; G - Disease-specific survival (DSS), univariate data; H - DSS, multivariate data; I - Recurrence-free survival (RFS), univariate data; J - RFS, multivariate data. The horizontal axis and the vertical axis represent the hazard ratio (HR) and the standard error of the effect size, respectively.
